# Causal Metabolomic and Lipidomic Analysis of Circulating Plasma Metabolites in Autism: A Comprehensive Mendelian Randomization Study with Independent Cohort Validation

**DOI:** 10.3390/metabo14100557

**Published:** 2024-10-17

**Authors:** Zhifan Li, Yanrong Li, Xinrong Tang, Abao Xing, Jianlin Lin, Junrong Li, Junjun Ji, Tiantian Cai, Ke Zheng, Sai Sachin Lingampelly, Kefeng Li

**Affiliations:** 1 Big Data and Internet of Things Program, Faculty of Applied Sciences, Macao Polytechnic University, Macao 999078, China; p2311274@mpu.edu.mo (Z.L.); p2213983@mpu.edu.mo (J.L.); p2311293@mpu.edu.mo (T.C.); p2316188@mpu.edu.mo (K.Z.); 2 Center for Artificial Intelligence-Driven Drug Discovery, Faculty of Applied Sciences, Macao Polytechnic University, Macao 999078, China; p2314602@mpu.edu.mo (Y.L.); p2314405@mpu.edu.mo (A.X.); jianlinlin@stu.xmu.edu.cn (J.L.); jijunjun@czmc.edu.cn (J.J.); 3 Yantai Special Education School, Yantai 264001, China; tangxinrong1212@163.com; 4 Department of Medicine, University of California, San Diego School of Medicine, San Diego, CA 92103-8467, USA; sachinlingampally@gmail.com

**Keywords:** autism spectrum disorder, metabolites, Mendelian randomization, causal inference, cohort validation, machine learning

## Abstract

Background: The increasing prevalence of autism spectrum disorder (ASD) highlights the need for objective diagnostic markers and a better understanding of its pathogenesis. Metabolic differences have been observed between individuals with and without ASD, but their causal relevance remains unclear. Methods: Bidirectional two-sample Mendelian randomization (MR) was used to assess causal associations between circulating plasma metabolites and ASD using large-scale genome-wide association study (GWAS) datasets—comprising 1091 metabolites, 309 ratios, and 179 lipids—and three European autism datasets (PGC 2015: *n* = 10,610 and 10,263; 2017: *n* = 46,351). Inverse-variance weighted (IVW) and weighted median methods were employed, along with robust sensitivity and power analyses followed by independent cohort validation. Results: Higher genetically predicted levels of sphingomyelin (SM) (d17:1/16:0) (OR, 1.129; 95% CI, 1.024–1.245; *p* = 0.015) were causally linked to increased ASD risk. Additionally, ASD children had higher plasma creatine/carnitine ratios. These MR findings were validated in an independent US autism cohort using machine learning analysis. Conclusion: Utilizing large datasets, two MR approaches, robust sensitivity analyses, and independent validation, our novel findings provide evidence for the potential roles of metabolomics and circulating metabolites in ASD diagnosis and etiology.

## 1. Introduction

Autism spectrum disorder (ASD) is considered a multifaceted neurodevelopmental disorder. The prevalence of ASD has shown a striking upward trend in recent decades, a phenomenon that has garnered considerable attention [1]. Individuals with ASD are characterized by impaired social interactions, communication difficulties, and repetitive patterns of behavior, with symptoms that usually appear before the age of three and persist throughout life, placing a profound burden on society and the individual [2,3].

The etiopathogenesis of ASD remains elusive [4] but burgeoning genetic and molecular biological evidence points to a syncretism of heritable and environmental factors in its pathophysiology [5,6].

Recently, the association between ASD and blood metabolites has garnered significant scientific interest. Metabolites—small molecules involved in cellular processes as substrates, intermediates, and end products, encompassing lipids, amino acids, carbohydrates, and vitamins—not only reflect the body’s physiological and pathological states but also serve as critical links between genetic makeup and phenotypic manifestations [7]. An increasing number of observational studies have identified marked metabolic disparities in individuals with ASD compared to healthy counterparts, suggesting their potential roles in ASD-related deficits in brain function and neurodevelopment [8].

Early intervention is crucial for modifying social and behavioral trajectories in ASD, making the quest for early detection indispensable [9,10]. Timely intervention during the critical stages of cognitive maturation holds promise for enhancing neurodevelopment and behavioral aptitudes, thereby enriching the lives of children with ASD and their families. Elucidating the metabolic changes in ASD could unveil etiologies, provide early diagnostic biomarkers, and expedite intervention. Moreover, it could pave the way for novel therapeutic targets, steering us toward safer and more precise treatment modalities.

Genome-wide association studies (GWASs), which identify genetic variants linked to diseases or traits, have become useful tools for dissecting complex disease genetics and uncovering the genetic underpinnings of various conditions [11]. By comparing genomic information between affected and control groups, a GWAS pinpoints the single nucleotide polymorphisms (SNPs) implicated in disease susceptibility.

Both metabolites and genetic variation can provide valuable clues for exploring the etiology of ASD. On one hand, metabolites are products of cellular processes that reflect physiological and pathological states, thus revealing biological abnormalities associated with ASD. On the other hand, SNPs are closely associated with disease and enable the delineation of genetic risk factors underpinning ASD. The synergistic utilization of metabolites and SNPs through an integrated multi-omics approach may provide a new perspective for understanding the complex etiopathogenesis of ASD. The recent development of genomics has facilitated the application of (Mendelian randomization) MR analysis. Grounded in Mendel’s genetic principles, MR utilizes genetic variants to assess causal relationships between exposures and outcomes. By emulating the randomization inherent in clinical trials, MR employs SNPs as instrumental variables for exposure, providing a robust level of causal inference.

This study aimed to elucidate the causal association between ASD and the circulating plasma metabolome and lipidome using bidirectional two-sample MR methods. To ensure the robustness of our results, we performed two different MR methods and conducted a series of sensitivity analyses. Furthermore, we conducted an independent validation using an autism cohort from the United States. In quantifying this relationship, our objectives are twofold: (1) to enhance the prospects of early detection and intervention for ASD, significantly improving clinical outcomes for individuals with ASD and related neurodevelopmental disorders, and (2) to provide novel insights into the metabolic underpinnings of ASD, which could pave the way for the development of targeted therapeutic strategies and personalized medicine approaches.

## 2. Materials and Methods

**Figure 1:**
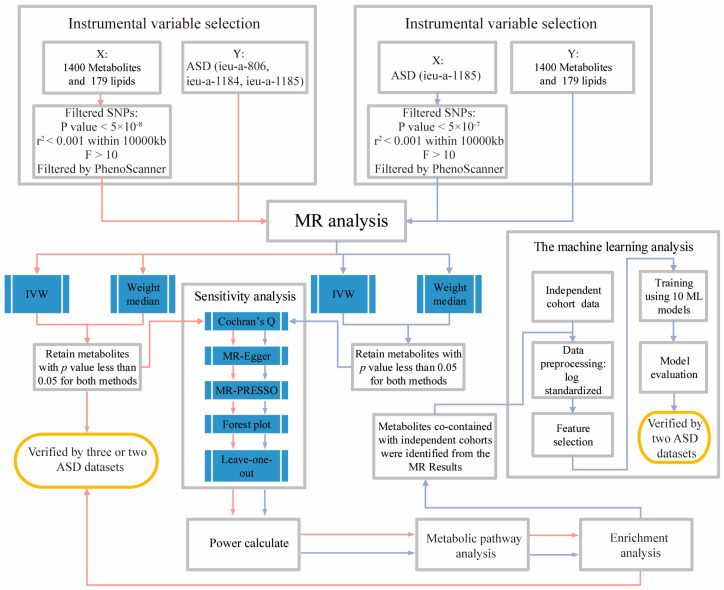
The entire flowchart of the experiment. Bidirectional two-sample MR analysis was used to explore the causal relationships between metabolites and ASD. Causal estimates in the MR analysis were considered statistically significant if the inverse-variance weighted (IVW) results yielded a *p* < 0.05, accompanied by weighted median results with a *p* < 0.05. In order to ensure the reliability of the study results, we performed a series of sensitivity analyses and calculated the power. In our reverse MR analysis, in order to prevent the unreliable results of a single ASD dataset, we also used a separate queue of another ASD dataset and added machine learning methods for validation. The results of forward MR were corroborated by at least two ASD datasets among ieu-a-806, ieu-a-1184, and ieu-a-1185. The reverse MR findings were validated using the ieu-a-1185 dataset and an independent cohort. Abbreviations: IVW: Inverse-variance weighted; r2: R-squared. The extent to which genetic variation explains the variation in exposure factors (risk factors); F: F statistic. Red line: The forward MR analysis process. Blue line: The reverse MR analysis process.

The main methodological flow of this study is shown in Figure 1. Corresponding PubChem IDs for the key metabolites are detailed in Table S21, while Table S22 provides a comprehensive list of version numbers for all software utilized in this research.

### 2.1. Study Design and Data Sources

**Figure 2:**
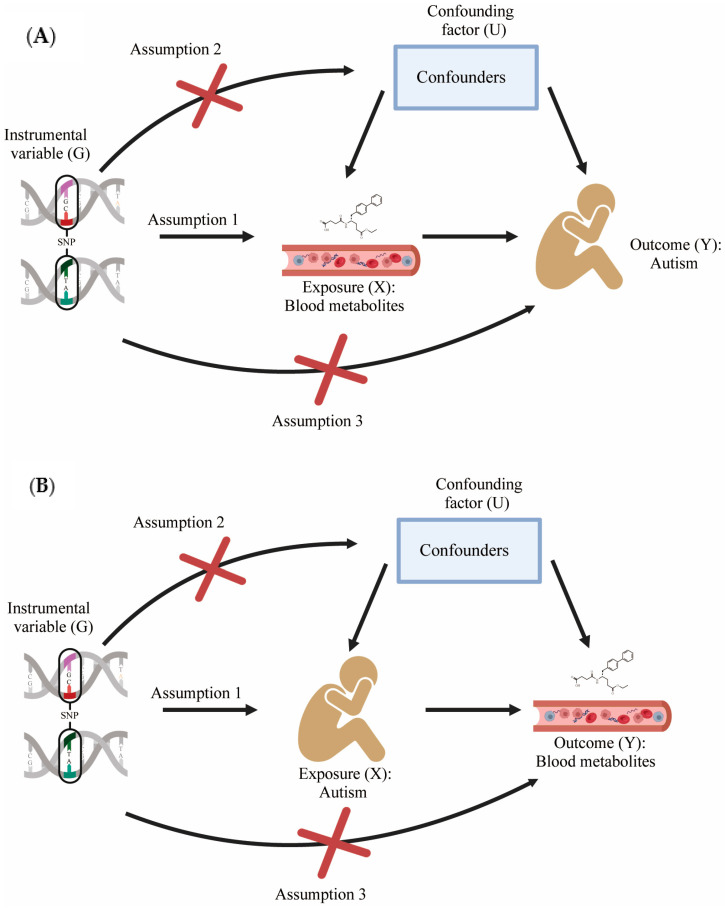
Three hypotheses of MR and rules for data sources and SNP screening. (**A**) Forward MR process. (**B**) Reverse MR process; SNPs: single-nucleotide polymorphisms. Assumption 1: Instrumental variables must be strongly correlated with exposure factor X. Assumption 2: Instrumental variables cannot be associated with any possible confounders. Assumption 3: Instrumental variables cannot be directly related to the outcome.

To explore the potential causal links between childhood autism and blood metabolites, we employed a bidirectional MR approach with a two-sample design. This robust epidemiological approach uses genetic variants as instrumental variables to assess the causal relationship between risk factors and health outcomes [12,13] (Figure 2). Our analysis incorporated blood metabolite data from a comprehensive GWAS led by Yiheng Chen et al. (2023) [7], examining 1091 blood metabolites and 309 metabolite ratios in 8299 unrelated European individuals from the Canadian Longitudinal Study on Aging (CLSA). We also included the lipidomic GWAS data of 179 lipids from 7174 Finnish participants—provided by Linda Ottensmann (2023) (Table S1 provides the summary information of metabolite data, and Tables S2 and S3 provide the list of metabolites used) [14].

The ASD-related data were sourced from three large-scale European autism genome-wide association studies (GWASs) conducted by the Psychiatric Genomics Consortium (PGC). These included two studies from 2015 with sample sizes of 10,610 and 10,263, respectively, and a larger study from 2017 with a sample size of 46,351 (Table S4 provides summary information of metabolite data).

To validate the reliability of the Mendelian randomization experiment’s results, we utilized the 5-year-old ASD dataset published by Sai Sachin Lingampelly (2024) [15]. This dataset comprises 53 participants, including 39 males and 15 females, with 31 individuals diagnosed with ASD and 22 serving as controls.

### 2.2. Instrumental Variables Selection

In forward MR analyses, SNPs associated with blood metabolites and lipid data were selected based on a stringent significance threshold (*p* < 5 × 10^−8^), which aligns with established GWAS criteria. To adjust for linkage disequilibrium (LD) and ensure independence among SNPs, we used PLINK v1.9 with pre-specified parameters (10,000 KB window size, r^2^ < 0.001) [16,17]. SNPs lacking ample statistical intensity (F-statistic < 10) were excluded to prevent weak instrument bias. Further scrutiny using PhenoScanner V2 allowed us to identify and eliminate SNPs that might confound the association with ASD [18]. In reverse MR analysis, ASD was treated as the exposure, and metabolites were treated as outcomes. Initially, a standard genome-wide significance level (*p* < 5 × 10^−8^) was considered, but the absence of surviving SNPs resulted in a criterion relaxation to a *p*-value of 5 × 10^−7^, permitting further examination and analyses (Figure 1).

### 2.3. Mendelian Randomization Analysis

For MR analysis, we utilized several methodologies, including inverse-variance weighted (IVW), weighted median, simple mode, weighted mode, and MR-Egger, with the random-effects IVW and weighted median methods serving as primary strategies. We interpreted causal relationships as significant when IVW and weighted median *p*-values both indicated statistical significance (*p* < 0.05) (Figure 1).

### 2.4. Sensitivity Analysis

Heterogeneity tests via Cochran’s Q metric and pleiotropy assessments through MR-Egger were incorporated into our sensitivity analyses [19,20]. The absence of significant heterogeneity (*p* > 0.05) in instrumental variables indicated uniformity across genetic variants, while a non-significant MR-Egger intercept (*p* > 0.05) suggested minimal pleiotropy. Forest plots generated via a leave-one-out approach highlighted influential SNPs, guiding their potential exclusion. Power calculations were also performed to estimate the adequacy of our instrument strength [21] (Figure 1).

### 2.5. Machine Learning Analysis

To investigate and validate the effect of autism on metabolites, we used significant results (IVW and WM *p* < 0.05) derived from previous MRs as the primary study then we looked for metabolites or metabolite ratios from the independent validation cohort that were identical to or directly correlated with the MR results via enrichment and metabolic pathway analyses. Subsequently, these features underwent a natural logarithm (log e) transformation, followed by normalization using the StandardScaler, which standardizes the features to a mean of 0 and a standard deviation of 1. After that, Spearman’s method was used to find the few features that are most relevant and have the smallest *p*-value for modeling and analysis.

We employed ten common machine learning algorithms for modeling, including logistic regression (LR), support vector machine (SVM), k-nearest neighbor (KNN), Gaussian naive Bayes (GaussianNB), latent Dirichlet allocation (LDA), multi-layer perceptron (MLP), random forest (RF), light gradient boosting machine (LightGBM), extreme gradient boosting (XGBoost), and decision trees (DTs). Among these, RF, LightGBM, and XGBoost are ensemble learning algorithms based on decision trees used as base learners. RF is based on bagging, while LightGBM and XGBoost are based on the gradient boosting algorithm. GaussianNB assumes that each feature follows a Gaussian distribution within each class and internally uses the Gaussian probability density function to calculate probabilities.

To ensure the robustness and generalizability of the model, we used a bootstrap-based approach as our main validation strategy, with the number of iterations set to 100, using 80% of the data as the training set and 20% of the data as the validation set each time. The default hyperparameters for the machine learning models were used in our analysis. Model performance was evaluated using several metrics, including accuracy, precision, recall, F1 score, and the area under the receiver operating characteristic curve (AUROC) (Figure 1).

## 3. Results

### 3.1. Results Description

#### 3.1.1. Causal Impact of Plasma Metabolites on Autism

Through the meticulous selection of instrumental variables, we identified significant SNPs to investigate the causal relationships between blood metabolites and the risk of ASD using MR analysis. The analysis focused on instances where both the IVW and weighted median methodologies yielded concurrent positive findings (Tables S5–S7 provide the results and OR values of the five MR methods for filtered metabolites, with the positive results for the lipid data in Table S17).

**Figure 3:**
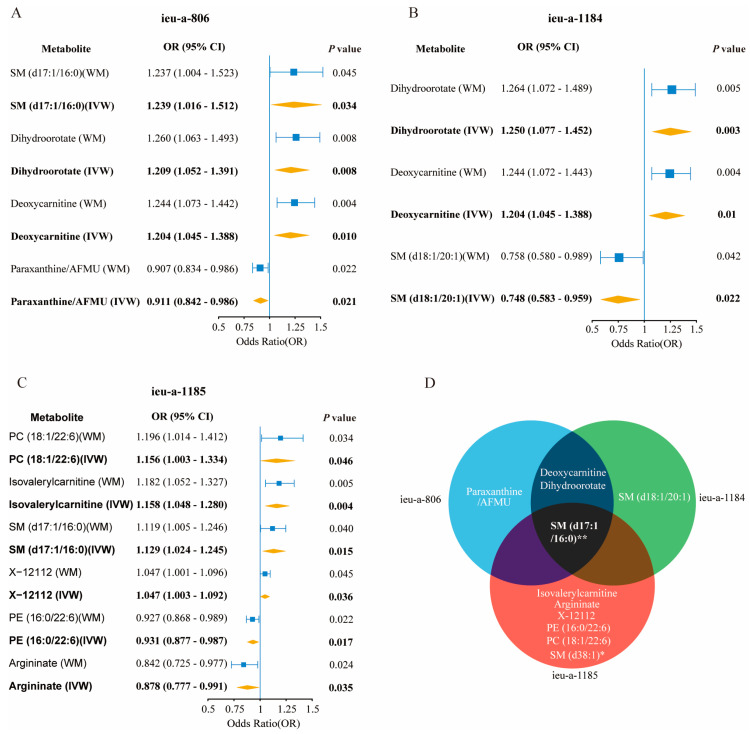
The genetically predicted changes in the plasma metabolite levels causally linked to autism. (**A**–**C**) The significant associations between plasma circulating metabolites and autism in the GWAS ieu-a-806 (**A**), ieu-a-1184 (**B**), and ieu-a-1185 (**C**) datasets, identified with two Mendelian randomization (MR) methods. The odds ratios represent the risk of developing autism compared to controls. Only metabolites with significant associations (*p* < 0.05) in both the inverse-variance weighted (IVW) and weighted median (WM) methods are shown. (**D**) A shared metabolite across three autism datasets, for which its genetically predicted concentration changes in plasma were associated with the occurrence of autism. *: 179 lipid group data for exposure, and the *p*-values of WM and IVW in the ieu-a-1185 dataset were both less than 0.05. **: The *p*-values of SM (d17:1/16:0) were <0.05 for both WM and IVW in the ieu-a-806 and ieu-a-1185 datasets, and they were significant only for the IVW method in the ieu-a-1184 dataset. The GWAS data for plasma metabolites were used as the exposure, and the ASD GWAS data were used as the outcomes. Abbreviations: WM: weighted median; IVW: inverse-variance weighted; SM: Sphingomyelin; AFMU: 5-acetylamino-6-formylamino-3-methyluracil; PC: phosphatidylcholine; X-12112: unidentified metabolite; PE: phosphatidylethanolamine.

- From all three analyzed ASD datasets, we observed several metabolites displaying a causal association with ASD (Figure 3). In the ieu-a-806 dataset, sphingomyelin (d17:1/16:0), dihydroorotate, and deoxycarnitine displayed a consistent positive causal relationship with ASD, while paraxanthine/AFMU exhibited an inverse association. In the ieu-a-1184 dataset, both dihydroorotate and deoxycarnitine also revealed a significant positive causal relationship with ASD, while SM (d18:1/20:1) was negatively associated. In ieu-a-1185, additional metabolites such as PC (18:1/22:6), isovalerylcarnitine, SM (d17:1/16:0), and X−12112 were positively linked to ASD, whereas PE (16:0/22:6) and argininate demonstrated a reverse causal relationship.

Evidently, deoxycarnitine emerged with a strong positive causality in both the ieu-a-806 and ieu-a-1184 datasets, with notable findings in ieu-a-1184: dihydroorotate [IVW: OR, 1.250; 95% CI, 1.077–1.452; *p* = 0.003] and deoxycarnitine [IVW: OR, 1.204; 95% CI 1.045–1.388; *p* = 0.010].

The consistency of the results for SM (d17:1/16:0) across the datasets was significant, particularly within ieu-a-1185 ([IVW: OR, 1.129; 95% CI, 1.024–1.245; *p* = 0.015]), emphasizing a potential causal effect on ASD risk.

**Figure 4:**
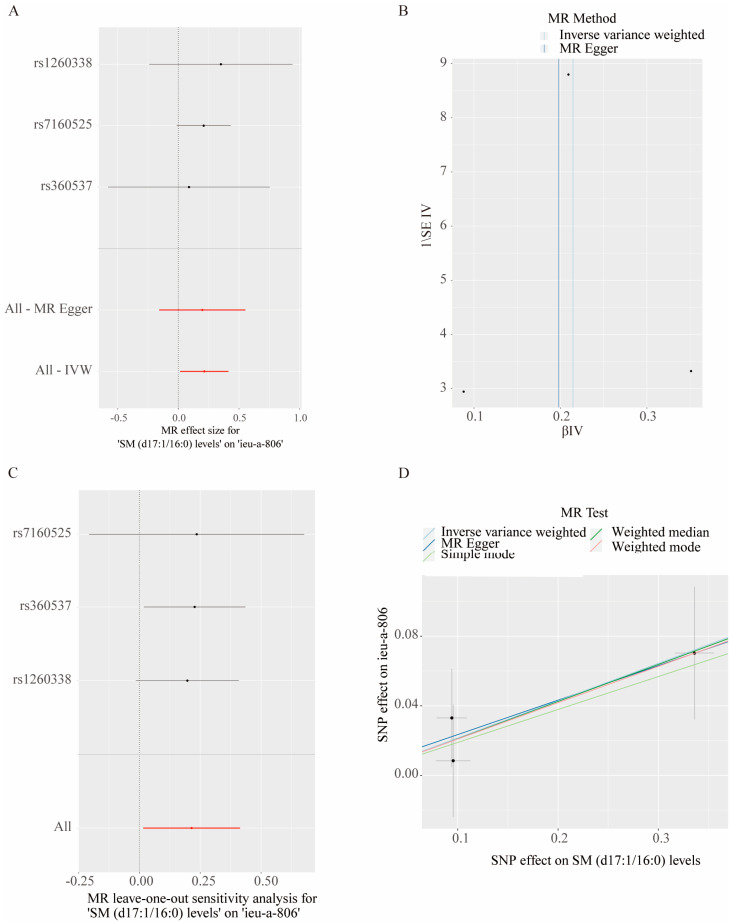
Various sensitivity analyses showed the robustness of the causal associations between metabolites and autism. (**A**) The forest plot shows no heterogeneity in causal effects amongst the instruments. Each black point represents the effect size for ASD per the standard deviation (SD) increase in the SM (d17:1/16:0), produced using each SNP as a separate instrument, and red points show the combined causal estimate using all SNPs together in a single instrument—using two different methods, including the inverse-variance weighted (IVW) and MR-Egger methods. Horizontal lines denote 95% confidence intervals. (**B**) The funnel plot shows the relationship between the causal effect of SM (d17:1/16:0) and ASD estimated using each individual SNP as a separate instrument against the inverse of the SE of the causal estimate. The vertical lines show the causal estimates using all the SNPs combined into a single instrument for each of the two different methods. Asymmetry in the funnel plot may be indicative of violations of the instrumental variable (IV) through horizontal pleiotropy. (**C**) The leave-one-out sensitivity analysis indicated that there are no instances in which the exclusion of one particular SNP leads to dramatic changes in the overall result. Each black point represents the IVW MR method applied to estimate the causal effect of SM (d17:1/16:0) on ASD, excluding that particular variant from the analysis. The red point depicts the IVW estimate using all the SNPs. (**D**) The scatter plot summarizes the MR estimates using the 5 methods of statistics. The β-value with the standard error (SE) is plotted to demonstrate the effect estimate of each single nucleotide polymorphism (SNP) for the causal association of SM (d17:1/16:0) (*x*-axis) with ASD (*y*-axis). The slope of each line represents the two-sample MR estimate (β-value) for the individual SNP. The error bar represents the SE of the effect size. SM (d17:1/16:0) was used as a representative exposure, and the autism GWAS ieu-a-806 dataset was used as a representative outcome.

Heterogeneity tests, as delineated by Cochran’s Q, validated the uniformity of the data without significant diversity (*p* > 0.05) (the heterogeneity results are provided in Tables S8–S10, while the results of the lipid data are presented in Table S18). Pleiotropy was excluded based on the MR-Egger results (*p* > 0.05) (Tables S11–S13, while the results of the lipid data are presented in Table S19). MR–PRESSO showed no horizontal pleiotropy. The leave-one-out analysis and forest plots confirmed the robustness of the findings, with no outliers necessitating exclusion (Figure 4) (various major sensitivity analyses are shown in Figures S1–S7).

#### 3.1.2. Causal Impact of Autism on Plasma Metabolites

**Figure 5:**
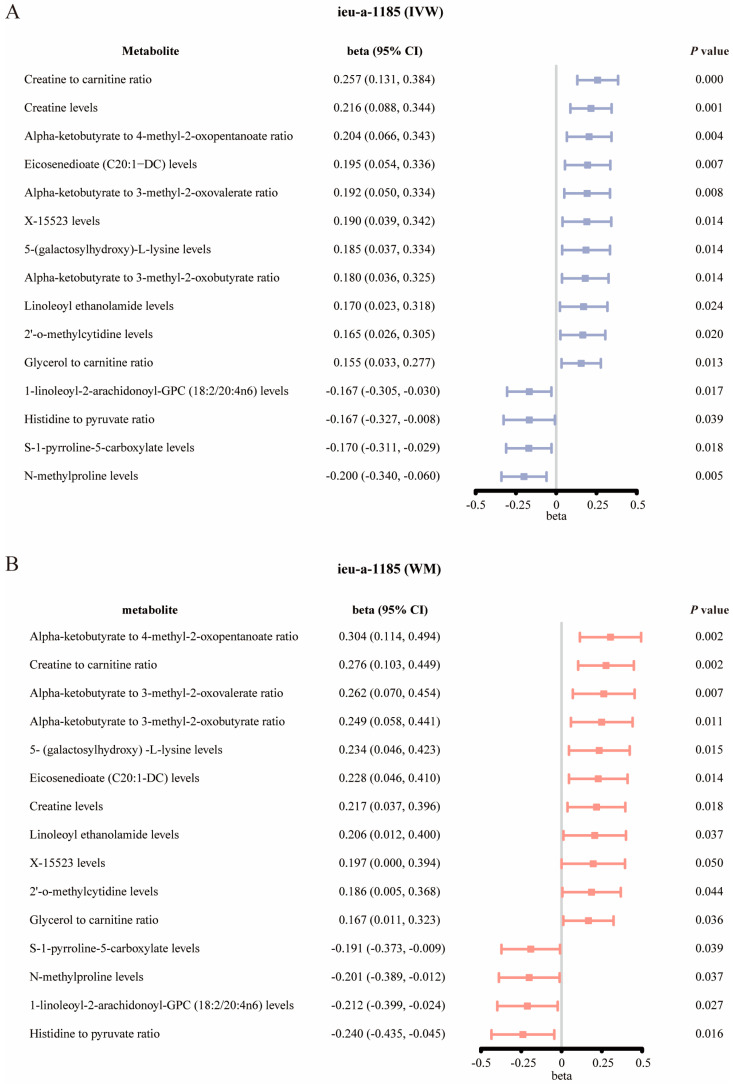
The changes in circulating plasma metabolites causally associated with genetically predicted autism. (**A**,**B**) The significant associations between genetically predicted autism (ieu-a-1185) and circulating plasma metabolites, identified using the inverse-variance weighted (IVW) random-effect method (**A**) and the weighted median method (**B**). Beta values (effect sizes) and 95% confidence intervals (CIs) indicate the magnitude of metabolite concentration changes in autism patients versus controls. A beta value < 0 denotes a decreased metabolite concentration. Abbreviation: GPC: glycerylphosphorylcholine.

The reverse MR analysis, with ASD as the exposure influencing blood metabolite levels, unveiled a causal relationship between ASD and 15 metabolites/metabolite ratios (Figure 5) (Table S14 shows the results and OR values of the five MR methods), all passing heterogeneity and horizontal pleiotropy assessments (*p* > 0.05) (Tables S15 and S16 show the results for heterogeneity detection and pleiotropy detection). Leave-one-out cross-validation detected no deviant data points (various major sensitivity analyses are shown in Figures S8–S12).

**Figure 6:**
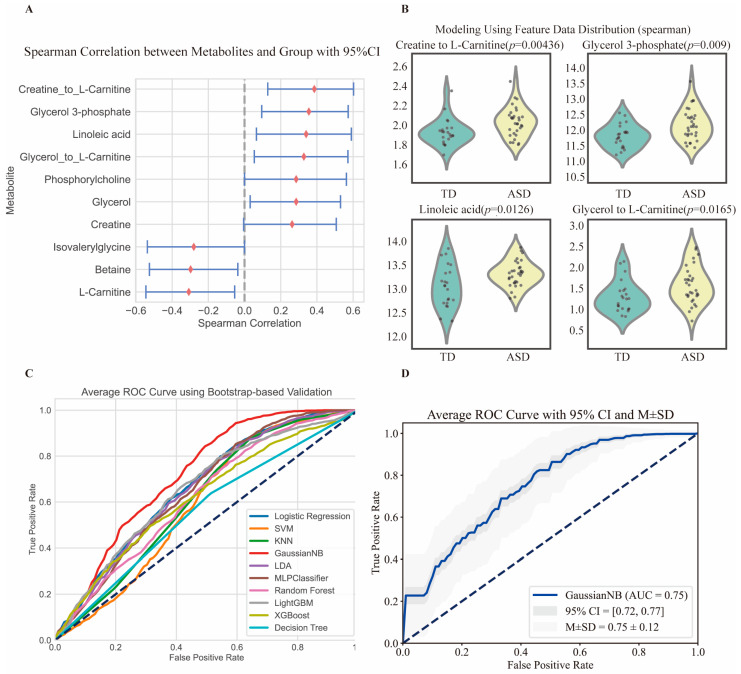
The validation of autism with the associated plasma circulating metabolites identified via MR analysis using an independent cohort. (**A**) Autism exhibited a robust correlation with specific plasma metabolites and their respective ratios. A Spearman rank correlation analysis was conducted to quantify these relationships, with the resulting correlation coefficients (r) and their corresponding 95% CI graphically represented. (**B**) The violin plots show the distribution of the four metabolites used in our final modeling between the ASD patients and controls, as well as the *p*-values. (**C**) The ROC comparison among ten ML models is shown, where the Gaussian naive Bayes (GaussianNB) ROC value is the highest. (**D**) The ROC curve analysis demonstrates good performance in differentiating autism from the control group using the plasma metabolites identified via MR analysis. The GaussianNB method was used for model construction. The raw metabolomics data were contributed by et al. [15]. The dataset includes 31 autistic children and 22 typically developing controls. Please refer to the previous publication for details on patient enrollment and mass spectrometry data collection.

To further validate and utilize these metabolites, four metabolites or metabolite ratios were selected as features from the initially identified 15 metabolites or metabolite ratios for model training in the independent cohort (Figure 6). These were linoleic acid, glycerol 3-phosphate, the creatine/L-carnitine ratio, and the glycerol/L-carnitine ratio. The creatine/L-carnitine ratio and glycerol/L-carnitine ratio were directly present in the Mendelian randomization (MR) results. Linoleic acid was associated with 1-linoleoyl-2-arachidonoyl-GPC and linoleoyl ethanolamide, which are derivatives or components of linoleic acid metabolism. Glycerol 3-phosphate is directly related to glycerol, reflecting glycerol metabolism. These four metabolites were chosen not only because of their strong correlation with the MR results but also because they have the lowest *p*-values when evaluated using Spearman’s method, and they exhibit the highest model performance with respect to the machine learning results(Figure 6).

Finally, using ten machine learning methods for prediction, the Gaussian naive Bayes (Gaussian NB) method showed the best performance overall with an AUC value of 0.75 (Figure 6) (Table S20) (Figures S13 and S14). Notably, the ratio of creatine/L-carnitine exhibited the highest significance in both the MR results and machine learning outcomes.

### 3.2. Power Analysis

We meticulously computed the statistical power for all outcomes deemed significant [21]. Our calculations revealed that in the analysis with autism as the outcome, deoxycarnitine exhibited a power exceeding 0.8 in both the ieu-a-806 and ieu-a-1184 datasets (0.82 and 0.84, respectively), while dihydroorotate reached a power of 0.89 in ieu-a-1184 (Table 1). In the analysis with autism as the exposure, all our metabolites of concern demonstrated a power of 1 or were close to 1 (Table 2).

### 3.3. Enrichment Analysis and Metabolic Pathway Analysis

**Figure 7:**
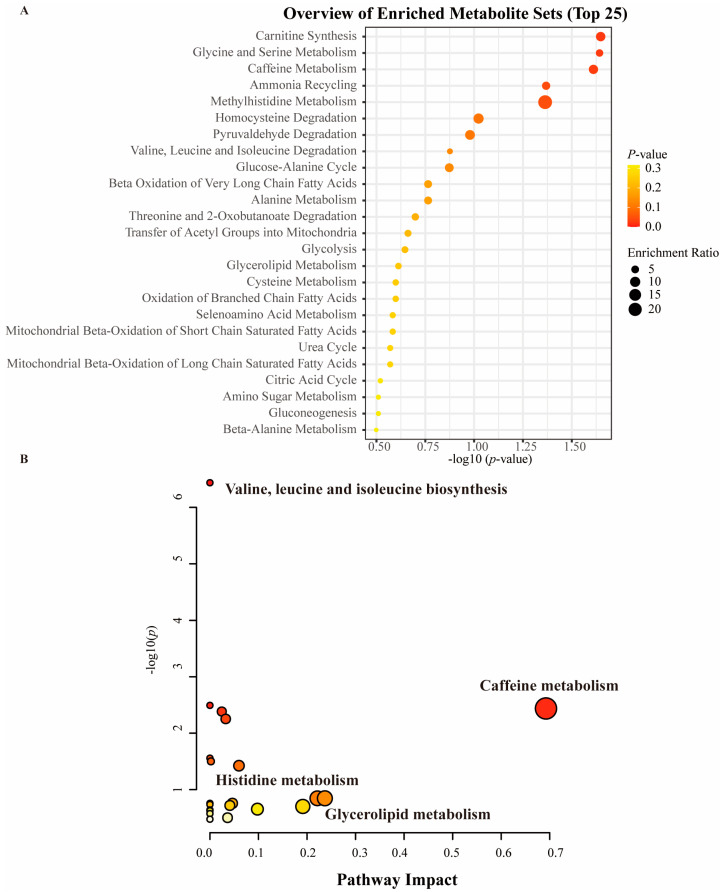
Enrichment analysis and pathway analysis of MR results. (**A**) Carnitine synthesis, glycine and serine metabolism, caffeine metabolism, and ammonia recycling exhibit significance in ASD. (**B**) Pathway analysis identified caffeine metabolism, glycerolipid metabolism, histidine metabolism, and valine, leucine, and isoleucine biosynthesis as significantly altered metabolic pathways in patients with ASD.

The results of our enrichment analysis show that carnitine synthesis, glycine and serine metabolism, caffeine metabolism, and ammonia recycling exhibit significant enrichment in autistic individuals and that these pathways work together and potentially cause neurodevelopmental problems, impaired energy metabolism, and neurotransmitter imbalances (Figure 7). Our pathway analyses identified caffeine metabolism; glycerolipid metabolism; histidine metabolism; and valine, leucine, and isoleucine biosynthesis as significantly altered metabolic pathways in patients with ASD (Figure 7).

## 4. Discussion

In our study, we applied MR to investigate the potential causative links between certain blood metabolites and ASD susceptibility. Our data highlighted that deoxycarnitine and dihydroorotate may have a direct causal effect on increasing ASD risk in two separate datasets, whereas SM (d17:1/16:0) was found to exhibit a consistent causal effect on elevating ASD risk across the three datasets. Having autism may have a potential causal effect on the creatine/carnitine ratio, which was further validated via the machine learning analysis of the independent cohorts.

**Figure 8:**
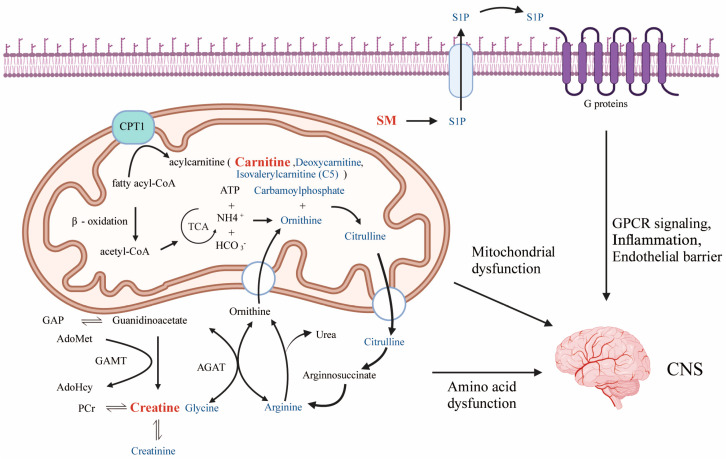
We identified in our study the primary metabolic pathways exhibiting alterations in autism, specifically noting changes in mitochondrial function, fatty acid metabolism, amino acid metabolism, creatine metabolism, and sphingolipid metabolism. Carnitine plays a pivotal role in facilitating the translocation of fatty acids into the mitochondrial matrix via the CPT1 system, where these substrates undergo β-oxidation for energy production. Perturbations in this process can lead to impaired mitochondrial function and compromised energy homeostasis. Creatine synthesis is catalyzed by the sequential actions of AGAT and GAMT. Disturbance of amino acid metabolism and related physiological processes in ASD patients may lead to deviations in creatine levels in the body. SM is an integral component of the S1P signaling pathway, which plays a pivotal role in regulating inflammation, GPCR signaling, and endothelial barrier integrity. Dysregulated SM levels can lead to inflammatory diseases, abnormal GPCR signaling cascades, and neuroinflammation. Abbreviations: SM: Sphingomyelin; CPT1: carnitine palmitoyltransferase; GAMT: guanidinoacetate N-methyltransferase; AGAT: arginine:glycine amidinotransferase; S1P: sphingosine-1-phosphate; GPCR: G protein-coupled receptor; CNS: central nervous system.

We identified that the primary metabolic pathways exhibiting alterations in ASD are related to mitochondrial function, fatty acid metabolism, amino acid metabolism, creatine metabolism, and sphingolipid metabolism (Figure 8). This is further supported by recent metabolomics studies showing significant changes in plasma, urine, and fecal metabolite profiles and dysregulation of mitochondrial function, oxidative stress, and amino acid metabolism-related pathways in ASD patients, which are consistent with our findings [8,22,23]. The observed metabolic perturbations in individuals with ASD may arise from remodelling and dysregulated protein and lipid turnover in tissues affected by ASD, particularly in the central nervous system (CNS).

These discoveries shed light on the potential metabolic mechanisms contributing to ASD and may pave the way for identifying novel biomarkers.

### 4.1. Sphingomyelin and Autism

Sphingomyelin is an essential phospholipid found in cell membranes, notably within neural tissues [24]. It is crucial for cell signaling, membrane structure, and cellular recognition. Its variants, such as SM (d17:1/16:0), all share a common structural feature: the presence of a sphingosine base and saturated fatty acids, resulting in a large molecule. Sphingomyelin metabolism produces sphingosine-1-phosphate (S1P), which plays a crucial role in neural development and synaptic functioning. Previous research has suggested that S1P metabolism may be disrupted in individuals with ASD [25,26].

Our study found a potential causal relationship between high levels of SM (d17:1/16:0) and ASD. The alterations in SM levels observed in individuals with ASD may reflect disturbances in protein and lipid metabolism in neural tissues. This metabolic remodelling could lead to disruptions in myelination, synaptic function, and overall neural connectivity. The SM metabolite, S1P, not only influences neurodevelopment and synaptic function but also regulates GPCR signaling, inflammatory responses, and endothelial barrier integrity. Dysregulation of the S1P signaling pathway might cause inflammatory disorders, aberrant GPCR cascades, and neuroinflammation, which could contribute to the pathophysiology of ASD by perturbing protein and lipid homeostasis in the central nervous system (Figure 8). Furthermore, sphingomyelin metabolism is closely linked to the metabolism of other lipids, such as ceramides and glycosphingolipids, which are crucial components of myelin sheaths and are essential for neuronal function and axonal integrity.

Our findings support the hypothesis that sphingomyelin and its metabolites are intricately connected to ASD, proposing new directions for ASD biomarker discovery and early intervention strategies, with sphingomyelin metabolism and S1P signaling providing fertile ground for therapeutic exploration.

### 4.2. Creatine/Carnitine Ratio and Autism

Our study reveals that the creatine/carnitine ratio is significantly elevated in individuals with ASD, and our enrichment analyses also showed significant changes in carnitine synthesis in autistic patients compared to healthy controls (Figure 7), indicating potential metabolic dysregulation in these patients.

Creatine and carnitine are generated in mammals after protein degradation, and their altered levels might serve as evidence of affected protein and lipid turnover in the CNS of individuals with ASD.

Creatine synthesis is driven by the sequential actions of arginine, AGAT, and GAMT (Figure 8). Disruptions in amino acid metabolism and related physiological processes in individuals with ASD may lead to altered creatine levels, particularly in the CNS, where creatine is crucial for energy metabolism in high-demand organs like the brain. Elevated creatine levels could signal reduced ATP production efficiency, with increased creatine synthesis acting as a compensatory mechanism against energy deficits—possibly due to dysregulated protein turnover and impaired energy metabolism in neural tissues.

Carnitine is essential for transporting long-chain fatty acids into the mitochondrial matrix via the CPT1 system, where they undergo β-oxidation to produce ATP (Figure 8). Dysregulation of this process impairs mitochondrial function and disrupts energy homeostasis, potentially affecting energy-demanding tissues such as the brain. Additionally, carnitine plays a role in mitigating oxidative stress. Deoxycarnitine, a key intermediate in carnitine biosynthesis, is fundamentally related to cellular energy homeostasis and the translocation of long-chain fatty acids into mitochondria for β-oxidation. Elevated blood levels of deoxycarnitine observed in individuals with ASD may reflect dysregulated protein and lipid metabolism in the CNS of these individuals. Observations of reduced plasma carnitine levels in ASD individuals and empirical evidence of improvement in ASD symptoms with carnitine supplementation underline the relevance of this pathway [27]. The reduced carnitine levels and elevated deoxycarnitine levels observed in patients with ASD could indicate impaired fatty acid metabolism and increased oxidative stress.

Abnormalities in carnitine, deoxycarnitine, and creatine are all linked to mitochondrial dysfunction and altered lipid turnover in the CNS. Measuring the ratio of creatine/carnitine can uncover metabolic processes that may go unnoticed when assessing single metabolites alone. This approach offers a more comprehensive understanding of mitochondrial dysfunction and disrupted lipid turnover in individuals with ASD.

This finding offers a potential direction for early intervention and treatment in ASD patients and provides insights into the pathophysiology of the disorder.

### 4.3. Dihydroorotate and Autism

In the biosynthesis pathway of pyrimidines, dihydroorotate serves as a critical intermediate, playing an essential role in the nucleic acid synthesis processes within humans and other organisms. This process predominantly relies on the catalytic action of dihydroorotate dehydrogenase (DHODH). Previous research has established that DHODH inhibitors can be utilized in the treatment of cancer and a variety of immune-related diseases [28]. A series of studies have indicated that abnormalities in the immune system may significantly impact the pathogenesis of autism [29,30,31].

Dihydroorotate is nestled within the pyrimidine synthesis pathway and is crucial for the formation of nucleic acids. Pyrimidine synthesis is one of the few biosynthetic pathways directly coupled to mitochondrial electron transport, meaning that a disruption in DHODH activity could also impair mitochondrial respiration and ATP production. Given the growing body of evidence suggesting mitochondrial dysfunction in ASD, it is possible that elevated dihydroorotate levels and altered DHODH activity may contribute to the mitochondrial abnormalities frequently observed in ASD patients.

The observed elevation in dihydroorotate related to an increased ASD risk in our study underscores the importance of nucleotide synthesis in neural development, and it signals DHODH as a promising target for ASD therapeutic intervention. In particular, DHODH inhibitors, which are already in clinical use for other conditions, may offer a promising avenue for modulating pyrimidine metabolism and potentially alleviating some of the metabolic and immune dysregulations associated with ASD.

### 4.4. Limitations

While our study provides contributions to understanding the metabolic associations with ASD, it is not without limitations. The use of European-specific data via Mendelian randomization may not reflect the intricate interplay of genetics and the environment observed in broader, more diverse populations. The sample size of the cohort data used for machine learning validation is not large enough, and heterogeneity between individuals may be substantial; moreover, the Gaussian NB algorithm easily results in overfitting when there is a strong correlation between features, which may lead to deviations in results. Furthermore, the complex network of metabolic interactions may not be fully represented by focusing on selected metabolites, potentially oversimplifying the metabolic dynamics associated with ASD. Our study, while providing valuable insights, may not capture the full complexity of these metabolic interactions. Finally, although we propose potential biomarkers and therapeutic targets, MR methods have many challenges for the interpretation of binary outcomes. Further research that is inclusive of diverse populations and clinical trials is essential in order to confirm the validity and effectively translate these findings into practical clinical interventions.

## 5. Conclusions

Employing a bidirectional two-sample MR approach and leveraging the most comprehensive metabolome and lipidome GWAS datasets known to us, this pioneering study provides compelling evidence for the causal roles of specific circulating plasma metabolites in ASD etiology. Our findings identify positive causal associations between elevated levels of SM (d17:1/16:0), deoxycarnitine, and dihydroorotate and increased ASD risk. Additionally, we establish a causal relationship between ASD risk and creatine/carnitine ratios, verified through an independent cross-country cohort analysis. The alterations observed in sphingomyelin, deoxycarnitine, and the creatine/carnitine ratios may stem from a common origin—dysregulated protein and lipid turnover in the CNS of individuals with ASD. These novel insights shed light on the metabolic underpinnings of ASD, offering vital clues for developing diagnostic biomarkers and early intervention strategies. While promising, future research should replicate these findings in larger cohorts and explore the underlying molecular mechanisms to guide personalized therapeutic approaches. By leveraging robust analyses, independent validation, and large-scale datasets, our study represents a significant stride toward unraveling the complex metabolic landscape of ASD and improving clinical outcomes for affected individuals.

## Figures and Tables

**Table 1:** Power analysis using metabolites as exposure and autism as outcome.

Metabolites	ASD Dataset	Sample Size Required	Power
SM (d17:1/16:0)	ieu-a-806	10,263	0.62
SM (d17:1/16:0)	ieu-a-1184	10,610	0.63
SM (d17:1/16:0)	ieu-a-1185	46,531	0.76
Deoxycarnitine	ieu-a-806	10,263	0.82
Deoxycarnitine	ieu-a-1184	10,610	0.84
Dihydroorotate	ieu-a-806	10,263	0.75
Dihydroorotate	ieu-a-1184	10,610	0.89
SM (d18:1/20:1)	ieu-a-1184	10,610	0.89
Paraxanthine/AFMU	ieu-a-806	10,263	0.61
Isovalerylcarnitine	ieu-a-1185	46,531	0.88
Argininate	ieu-a-1185	46,531	1
X-12112	ieu-a-1185	46,531	0.61
PE (16:0/22:6)	ieu-a-1185	46,531	0.73
PC (18:1/22:6)	ieu-a-1185	46,531	0.65
SM (d38:1)	ieu-a-1185	46,531	0.64

Abbreviation: SM: Sphingomyelins; AFMU: s5-acetylamino-6-formylamino-3-methyluracil; PE: phosphatidylethanolamine; PC: phosphatidylcholine; X-12112: unidentified metabolite.

**Table 2:** Autism as exposure and metabolites as outcome power values.

ASD Dataset	Metabolites	Sample Size (Outcome)	Power
ieu-a-1185	Creatine/carnitine ratio	8299	1
ieu-a-1185	Creatine	8299	1
ieu-a-1185	Alpha-ketobutyrate/4-methyl-2-oxopentanoate ratio	8299	1
ieu-a-1185	Eicosenedioate (C20:1-DC)	8299	1
ieu-a-1185	Alpha-ketobutyrate/3-methyl-2-oxovalerate ratio	8299	1
ieu-a-1185	X-15523	8299	1
ieu-a-1185	5-(galactosylhydroxy)-L-lysine	8299	1
ieu-a-1185	Alpha-ketobutyrate/3-methyl-2-oxobutyrate ratio	8299	1
ieu-a-1185	Linoleoyl ethanolamide	8299	0.99
ieu-a-1185	2′-o-methylcytidine	8299	1
ieu-a-1185	Glycerol/carnitine ratio	8299	0.99
ieu-a-1185	PC(18:2/20:4)	8299	1
ieu-a-1185	Histidine/pyruvate ratio	8299	1
ieu-a-1185	S-1-pyrroline-5-carboxylate	8299	1
ieu-a-1185	N-methylproline	8299	1

Abbreviation: PC: Phosphatidylcholine.

## Data Availability

The three autism datasets are from three IEU–Open GWAS projects ((https://gwas.mrcieu.ac.uk/datasets/ieu-a-806/ accessed on 28 November 2023), (https://gwas.mrcieu.ac.uk/datasets/ieu-a-1184/ accessed on 28 November 2023), and (https://gwas.mrcieu.ac.uk/datasets/ieu-a-1185/ accessed on 28 November 2023)), and the metabolite data are from the large-scale GWAS data released by Yiheng Chen et al. in 2023 (https://www.ebi.ac.uk/gwas/ accessed on 28 November 2023). Accession numbers for European GWASs: GCST90199621-90201020; lipidomic data from large-scale GWAS data released by Linda Ottensmann in 2023 (https://www.ebi.ac.uk/gwas/ accessed on 25 December 2023)—GWAS accession numbers are GCST90277238-GCST90277416. Five-year-old data from data released by Sai Sachin Lingampelly: https://www.nature.com/articles/s42003-024-06102-y accessed on 20 June 2024.

## Supplementary Materials

The following supporting information can be downloaded at https://www.mdpi.com/article/10.3390/metabo14100557/s1, Figure S1. Various sensitivity analyses showed the robustness of the causal association between dihydroorotate and autism spectrum disorder (ASD, GWAS ID: ieu-a-806); Figure S2. Various sensitivity analyses showed the robustness of the causal association between dihydroorotate and autism spectrum disorder (ASD, GWAS ID: ieu-a-1184); Figure S3. Various sensitivity analyses showed the robustness of the causal association between SM (d17:1/16:0) and autism spectrum disorder (ASD, GWAS ID: ieu-a-806); Figure S4. Various sensitivity analyses showed the robustness of the causal association between SM (d17:1/16:0) and autism spectrum disorder (ASD, GWAS ID: ieu-a-1184); Figure S5. Various sensitivity analyses showed the robustness of the causal association between SM (d17:1/16:0) and autism spectrum disorder (ASD, GWAS ID: ieu-a-1185); Figure S6. Various sensitivity analyses showed the robustness of the causal association between deoxycarnitine and autism spectrum disorder (ASD, GWAS ID: ieu-a-806); Figure S7. Various sensitivity analyses showed the robustness of the causal association between deoxycarnitine and autism spectrum disorder (ASD, GWAS ID: ieu-a-1184); Figure S8. Various sensitivity analyses showed the robustness of the causal association between autism spectrum disorder (ASD, GWAS ID: ieu-a-1185) and the creatine/carnitine ratio; Figure S9. Various sensitivity analyses showed the robustness of the causal association between autism spectrum disorder (ASD, GWAS ID: ieu-a-1185) and linoleoyl ethanolamide; Figure S10. Various sensitivity analyses showed the robustness of the causal association between autism spectrum disorder (ASD, GWAS ID: ieu-a-1185) and creatine; Figure S11. Various sensitivity analyses showed the robustness of the causal association between autism spectrum disorder (ASD, GWAS ID: ieu-a-1185) and the glycerol/carnitine ratio; Figure S12. Various sensitivity analyses showed the robustness of the causal association between autism spectrum disorder (ASD, GWAS ID: ieu-a-1185) and PC (18:2/20:4); Figure S13. ROC curves and 95% CI and mean ± SD for (A) decision tree, (B) GaussianNB, (C) KNN, (D) LDA, (E) LightGBM, and (F) logistic regression; Figure S14. ROC curves and 95% CI and mean ± SD for (A) MLP, (B) random forest, (C) SVM, and (D) XGBoost; Table S1: Source for the metabolome and lipidome GWAS dataset used in the MR analysis; Table S2. List of 1400 plasma metabolites used in this study; Table S3. List of 179 lipids used in this study; Table S4. Source for the ASD GWAS datasets; Table S5. ieu-a-806—both IVW and WM show significant OR results; Table S6. ieu-a-1184—both IVW and WM show significant OR results; Table S7. ieu-a-1185—both IVW and WM show significant OR results; Table S8. ieu-a-806 heterogeneity; Table S9. ieu-a-1184 heterogeneity; Table S10. ieu-a-1184 heterogeneity; Table S11. ieu-a-806 pleiotropy; Table S12. ieu-a-1184 pleiotropy; Table S13. ieu-a-1185 pleiotropy; Table S14. Reverse MR OR results—both IVW and WM are significant in ieu-a-1185; Table S15. Reverse MR ieu-a-1185 heterogeneity; Table S16. Reverse MR ieu-a-1185 pleiotropy; Table S17. Lipid dataset with ieu-a-1185 OR results—both IVW and WM are significant; Table S18. Lipid dataset with ieu-a-1185 heterogeneity; Table S19. Lipid dataset with ieu-a-1185 pleiotropy; Table S20: Machine learning algorithm performance comparison for distinguishing autism from the control group; Table S21: PubChem ID for all major metabolites; Table S22: Major software and version numbers used.
