# Supplementary material for: Causal Metabolomic and Lipidomic Analysis of Circulating Plasma Metabolites in Autism: A Comprehensive Mendelian Randomization Study with Independent Cohort Validation
Source: Metabolites. 2024 Oct 17;14(10):557. doi: 10.3390/metabo14100557 (PMC11509474; doi:10.3390/metabo14100557)
Supplement: Supplementary file 1 [file metabolites-14-00557-s001.zip › Supplemental_figures.pdf]

Supplemental Figures for

Causal metabolomic and lipidomic analysis of circulating plasma metabolites in autism: A comprehensive Mendelian randomization study with independent cohort validation

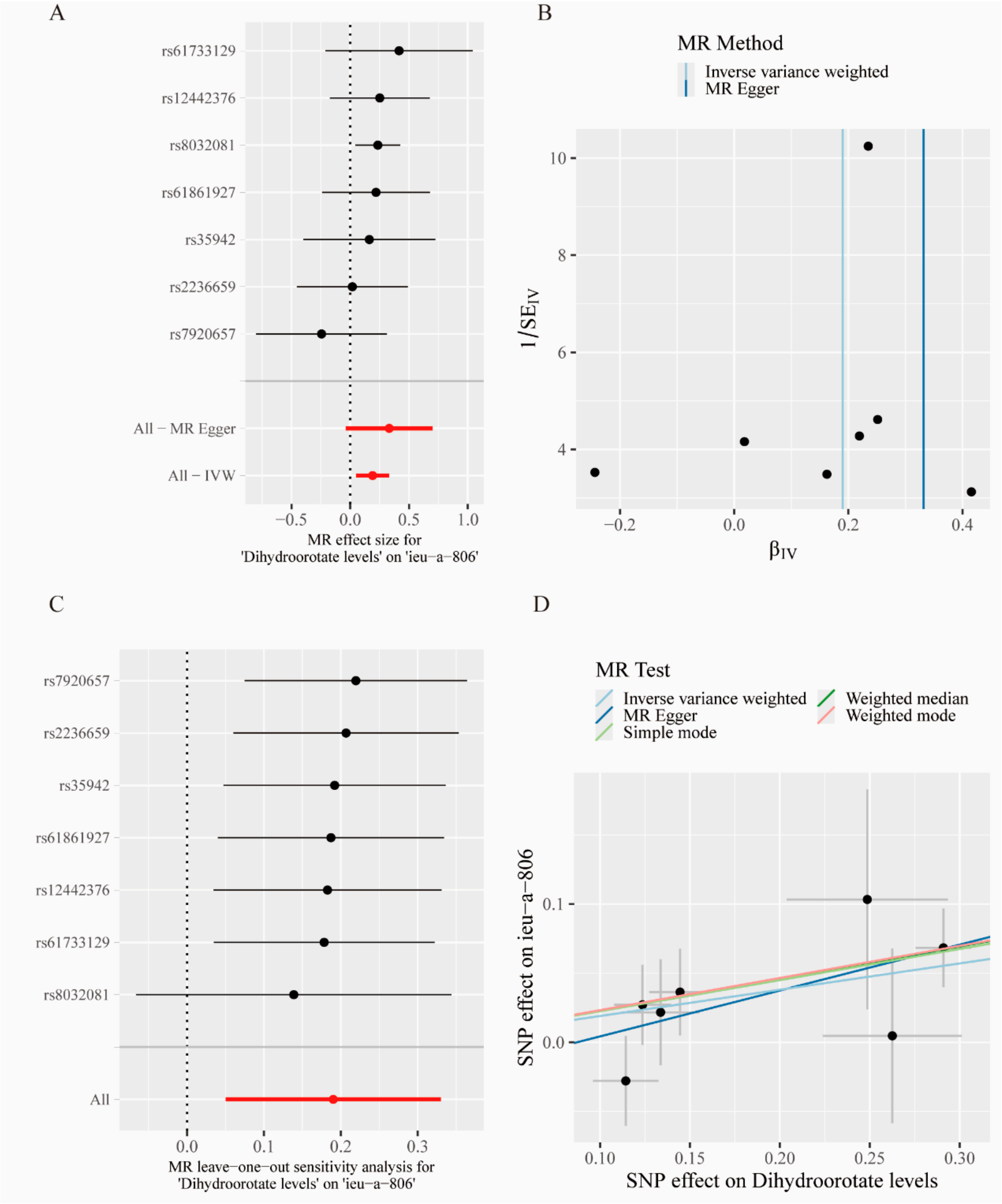

Figure S1. Various sensitivity analyses showed the robustness of the causal association between Dihydroorotate and Autism Spectrum Disorder (ASD, GWAS ID: ieu-a-806).

(A) The forest plot showing no heterogeneity in causal effects amongst instruments. Each black point represents the effect size for ASD per standard deviation (SD) increase in the Dihydroorotate produced using each of the SNPs as separate instruments, and red points showing the combined causal estimate using all SNPs together in a single instrument, using two different methods, including inverse variance weighted (IVW) and MR Egger. Horizontal lines denote 95% confidence intervals.

(B) Funnel plot showing the relationship between the causal effect of Dihydroorotate on the

ASD estimated using each individual SNP as a separate instrument against the inverse of the SE of the causal estimate. Vertical lines show the causal estimates using all SNPs combined into a single instrument for each of two different methods. Asymmetry in the funnel plot may be indicative of violations of the instrumental variable (IV) through horizontal pleiotropy.

(C) Leave one out sensitivity analysis indicated that there are no instances where the exclusion of one particular SNP leads to dramatic changes in the overall result. Each black point represents the IVW MR method applied to estimate the causal effect of Dihydroorotate on the ASD excluding that particular variant from the analysis. The red point depicts the IVW estimate using all SNPs.

(D) The scatter plot summarizing the MR estimates using 5 methods of statistics. The β-value with standard error (SE) is plotted to demonstrate effect estimate of each single nucleotide polymorphism (SNP) for causal association of Dihydroorotate (x-axis) with the ASD (y-axis). The slope of each line represents the two-sample MR estimate (β-value) for the individual SNP. Error bar represents SE of effect size.

Metabolite is the exposure and ASD is the outcome.

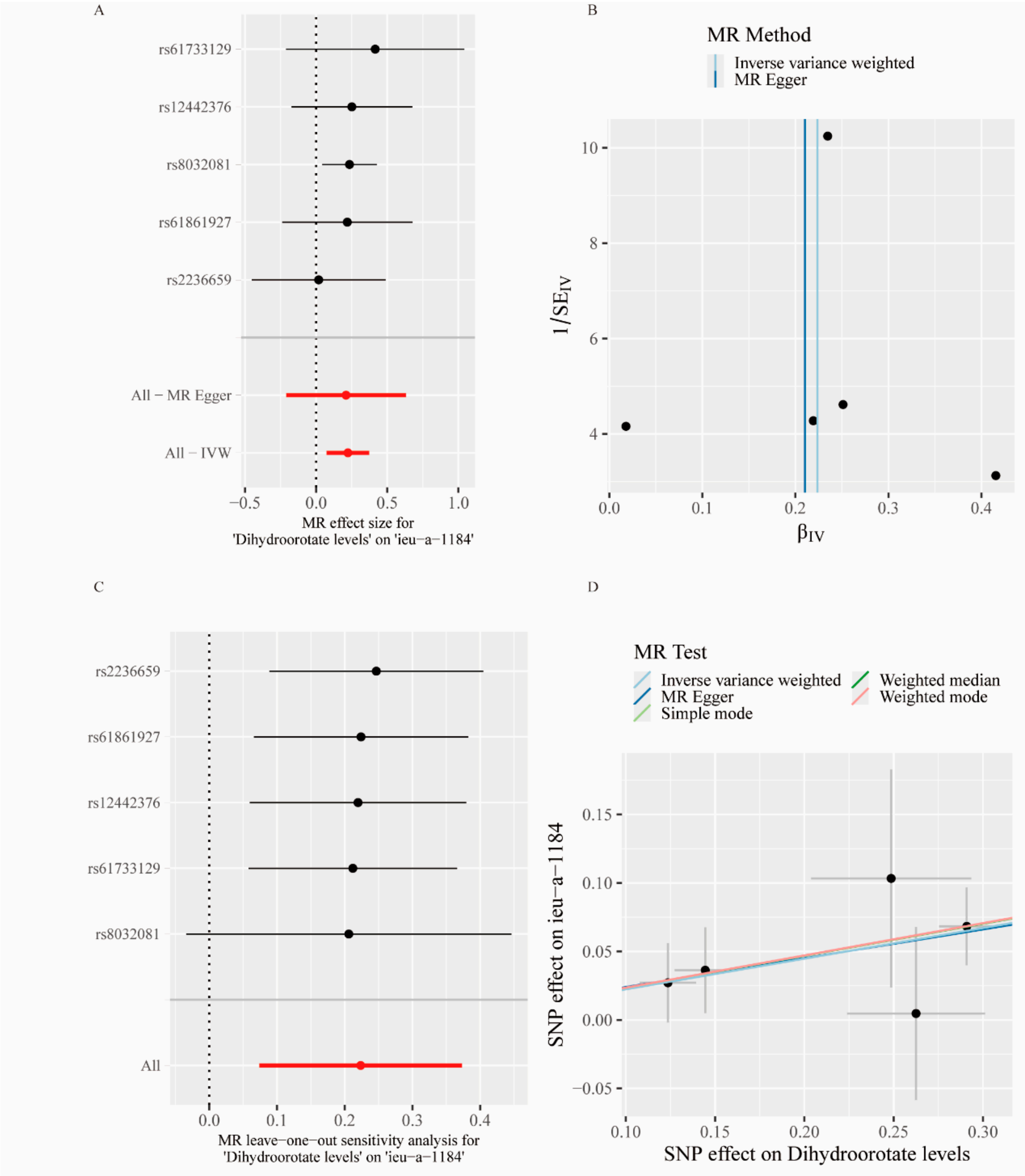

Figure S2. Various sensitivity analyses showed the robustness of the causal association between Dihydroorotate and Autism Spectrum Disorder (ASD, GWAS ID: ieu-a-1184).

(A) The forest plot showing no heterogeneity in causal effects amongst instruments. Each black point represents the effect size for ASD per standard deviation (SD) increase in the Dihydroorotate produced using each of the SNPs as separate instruments, and red points showing the combined causal estimate using all SNPs together in a single instrument, using two different methods, including inverse variance weighted (IVW) and MR Egger. Horizontal lines denote 95% confidence intervals.

(B) Funnel plot showing the relationship between the causal effect of Dihydroorotate on the ASD estimated using each individual SNP as a separate instrument against the inverse of the SE of the causal estimate. Vertical lines show the causal estimates using all SNPs combined into a single instrument for each of two different methods. Asymmetry in the funnel plot may be indicative of violations of the instrumental variable (IV) through horizontal pleiotropy.

(C) Leave one out sensitivity analysis indicated that there are no instances where the exclusion of one particular SNP leads to dramatic changes in the overall result. Each black point represents the IVW MR method applied to estimate the causal effect of Dihydroorotate on the ASD excluding that particular variant from the analysis. The red point depicts the IVW estimate using all SNPs.

(D) The scatter plot summarizing the MR estimates using 5 methods of statistics. The β-value with standard error (SE) is plotted to demonstrate effect estimate of each single nucleotide polymorphism (SNP) for causal association of Dihydroorotate (x-axis) with the ASD (y-axis). The slope of each line represents the two-sample MR estimate (β-value) for the individual SNP. Error bar represents SE of effect size.

Metabolite is the exposure and ASD is the outcome.

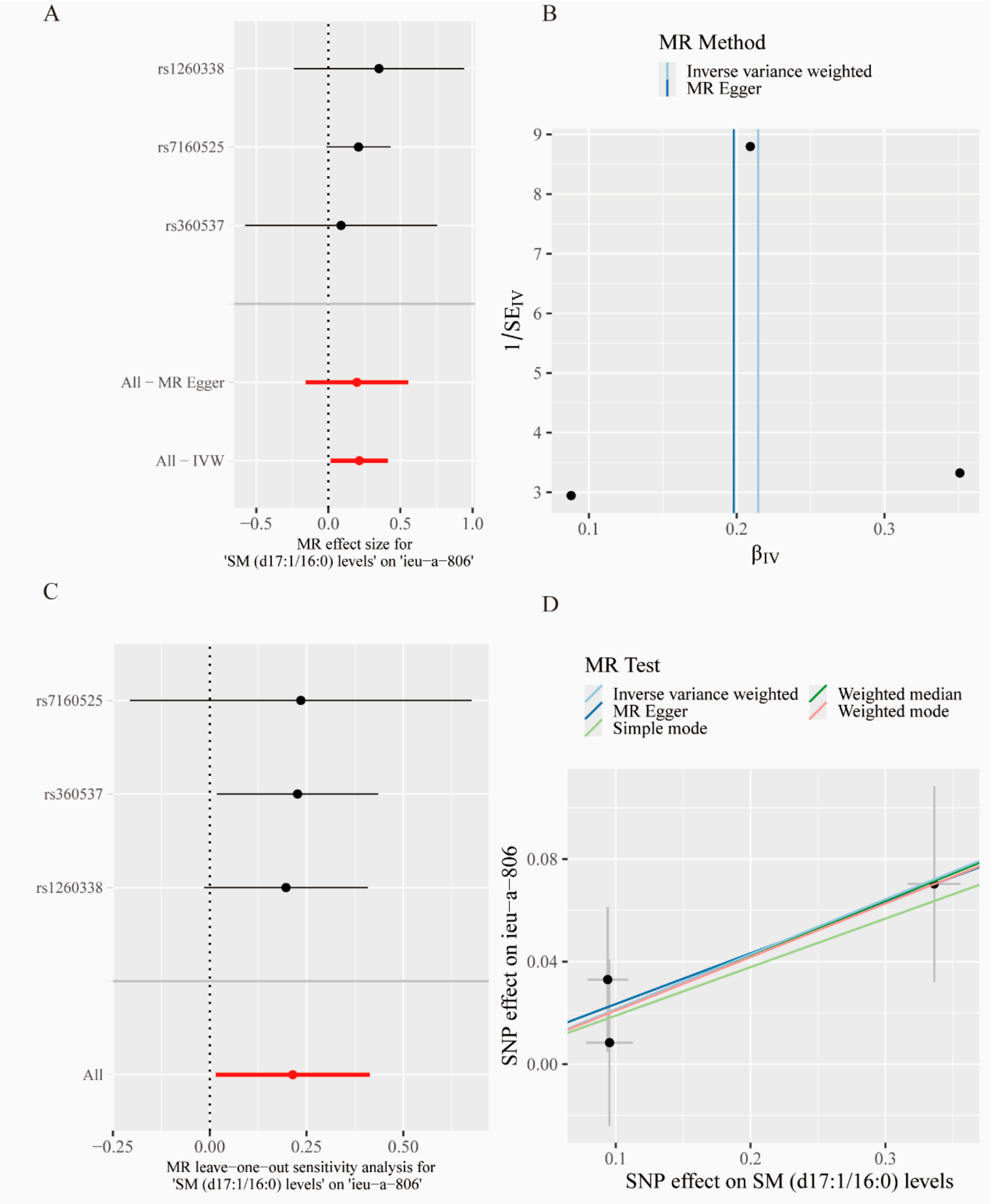

Figure S3. Various sensitivity analyses showed the robustness of the causal association between SM(d17:1/16:0) and Autism Spectrum Disorder (ASD, GWAS ID: ieu-a-806).
(A) The forest plot showing no heterogeneity in causal effects amongst instruments. Each black point represents the effect size for ASD per standard deviation (SD) increase in the SM(d17:1/16:0) produced using each of the SNPs as separate instruments, and red points showing the combined causal estimate using all SNPs together in a single instrument, using two different methods, including inverse variance weighted (IVW) and MR Egger. Horizontal lines denote 95% confidence intervals.
(B) Funnel plot showing the relationship between the causal effect of SM(d17:1/16:0) on the ASD estimated using each individual SNP as a separate instrument against the inverse of the SE of the causal estimate. Vertical lines show the causal estimates using all SNPs combined into a single instrument for each of two different methods. Asymmetry in the funnel plot may be indicative of violations of the instrumental variable (IV) through horizontal pleiotropy.
(C) Leave one out sensitivity analysis indicated that there are no instances where the exclusion of one particular SNP leads to dramatic changes in the overall result. Each black point represents the IVW MR method applied to estimate the causal effect of SM(d17:1/16:0) on the ASD excluding that particular variant from the analysis. The red point depicts the IVW estimate using all SNPs.
(D) The scatter plot summarizing the MR estimates using 5 methods of statistics. The β-value with standard error (SE) is plotted to demonstrate effect estimate of each single nucleotide polymorphism (SNP) for causal association of SM(d17:1/16:0) (x-axis) with the ASD (y-axis). The slope of each line represents the two-sample MR estimate (β-value) for the individual SNP. Error bar represents SE of effect size.
Metabolite is the exposure and ASD is the outcome.

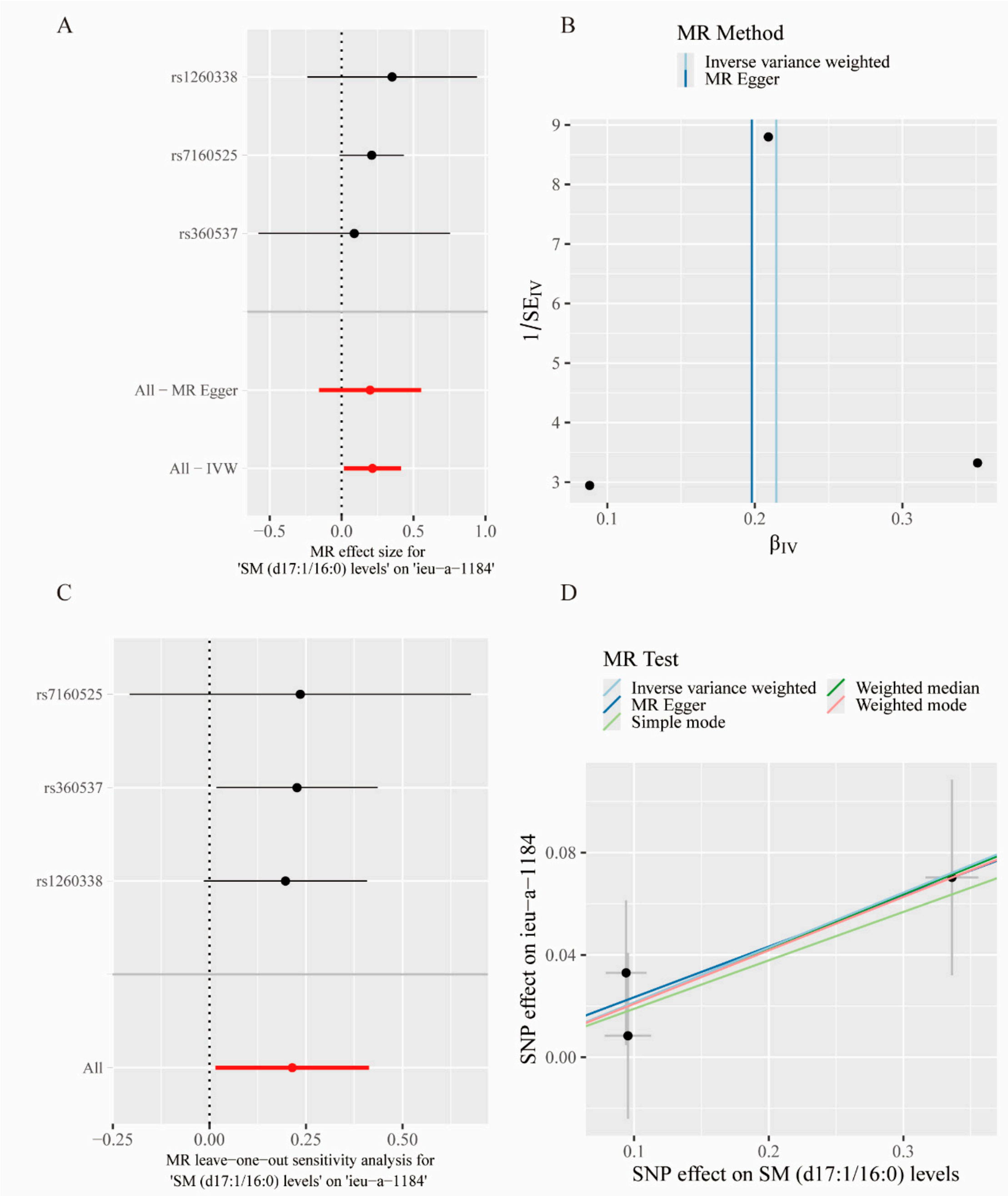

Figure S4. Various sensitivity analyses showed the robustness of the causal association between SM(d17:1/16:0) and Autism Spectrum Disorder (ASD, GWAS ID: ieu-a-1184).

(A) The forest plot showing no heterogeneity in causal effects amongst instruments. Each black point represents the effect size for ASD per standard deviation (SD) increase in the SM(d17:1/16:0) produced using each of the SNPs as separate instruments, and red points showing the combined causal estimate using all SNPs together in a single instrument, using two different methods, including inverse variance weighted (IVW) and MR Egger. Horizontal lines denote 95% confidence intervals.

(B) Funnel plot showing the relationship between the causal effect of SM(d17:1/16:0) on the ASD estimated using each individual SNP as a separate instrument against the inverse of the SE of the causal estimate. Vertical lines show the causal estimates using all SNPs combined into a single instrument for each of two different methods. Asymmetry in the funnel plot may

be indicative of violations of the instrumental variable (IV) through horizontal pleiotropy.

(C) Leave one out sensitivity analysis indicated that there are no instances where the exclusion of one particular SNP leads to dramatic changes in the overall result. Each black point represents the IVW MR method applied to estimate the causal effect of SM(d17:1/16:0) on the ASD excluding that particular variant from the analysis. The red point depicts the IVW estimate using all SNPs.

(D) The scatter plot summarizing the MR estimates using 5 methods of statistics. The β-value with standard error (SE) is plotted to demonstrate effect estimate of each single nucleotide polymorphism (SNP) for causal association of SM(d17:1/16:0) (x-axis) with the ASD (y-axis). The slope of each line represents the two-sample MR estimate (β-value) for the individual SNP. Error bar represents SE of effect size.

Metabolite is the exposure and ASD is the outcome.

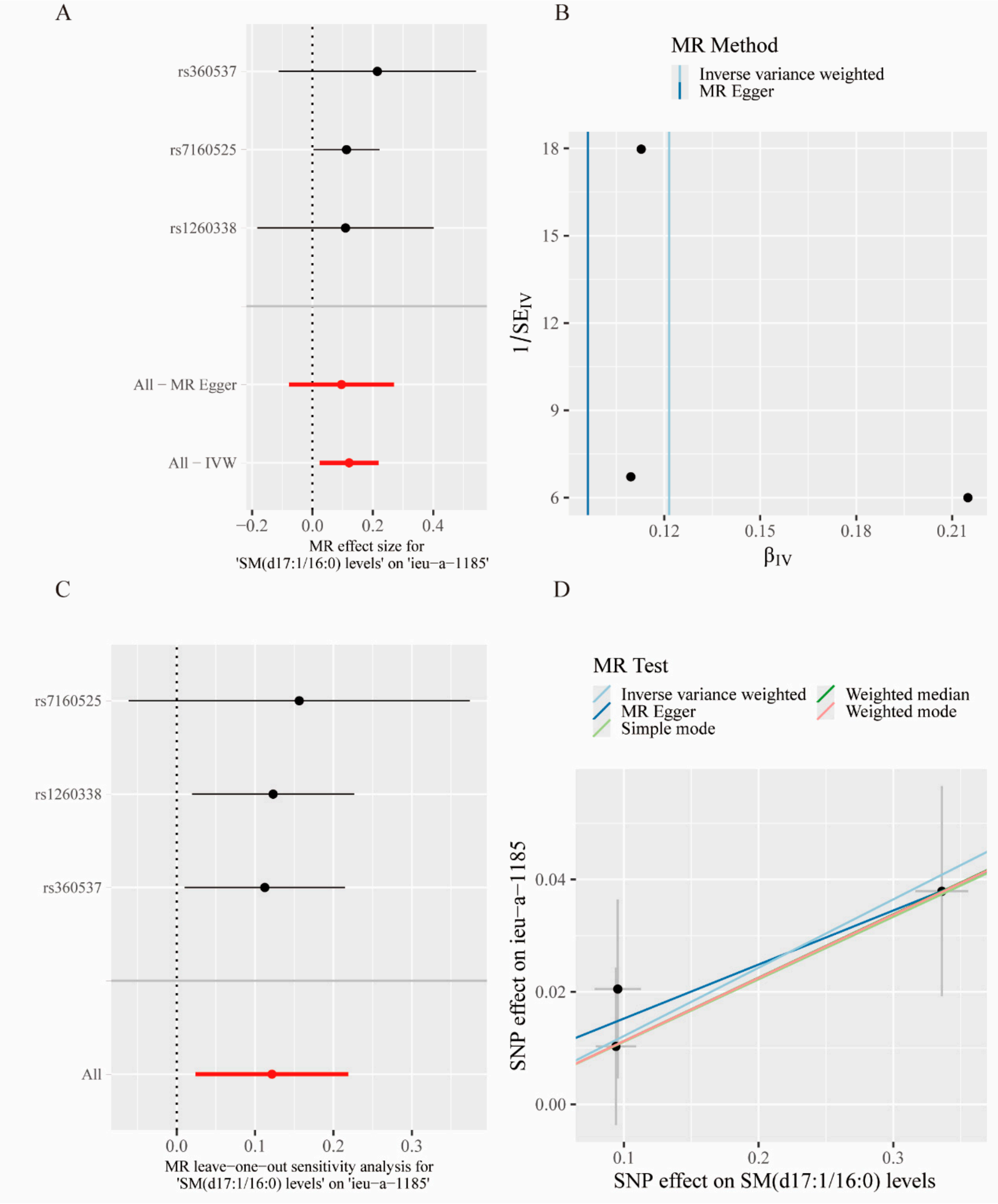

Figure S5. Various sensitivity analyses showed the robustness of the causal association between SM(d17:1/16:0) and Autism Spectrum Disorder (ASD, GWAS ID: ieu-a-1185).

(A) The forest plot showing no heterogeneity in causal effects amongst instruments. Each black point represents the effect size for ASD per standard deviation (SD) increase in the SM(d17:1/16:0) produced using each of the SNPs as separate instruments, and red points showing the combined causal estimate using all SNPs together in a single instrument, using two different methods, including inverse variance weighted (IVW) and MR Egger. Horizontal lines denote 95% confidence intervals.

(B) Funnel plot showing the relationship between the causal effect of SM(d17:1/16:0) on the ASD estimated using each individual SNP as a separate instrument against the inverse of the SE of the causal estimate. Vertical lines show the causal estimates using all SNPs combined into a single instrument for each of two different methods. Asymmetry in the funnel plot may be indicative of violations of the instrumental variable (IV) through horizontal pleiotropy.

(C) Leave one out sensitivity analysis indicated that there are no instances where the exclusion of one particular SNP leads to dramatic changes in the overall result. Each black point represents the IVW MR method applied to estimate the causal effect of SM(d17:1/16:0) on the ASD excluding that particular variant from the analysis. The red point depicts the IVW estimate using all SNPs.

(D) The scatter plot summarizing the MR estimates using 5 methods of statistics. The β-value with standard error (SE) is plotted to demonstrate effect estimate of each single nucleotide polymorphism (SNP) for causal association of SM(d17:1/16:0) (x-axis) with the ASD (y-axis). The slope of each line represents the two-sample MR estimate (β-value) for the individual SNP. Error bar represents SE of effect size.

Metabolite is the exposure and ASD is the outcome.

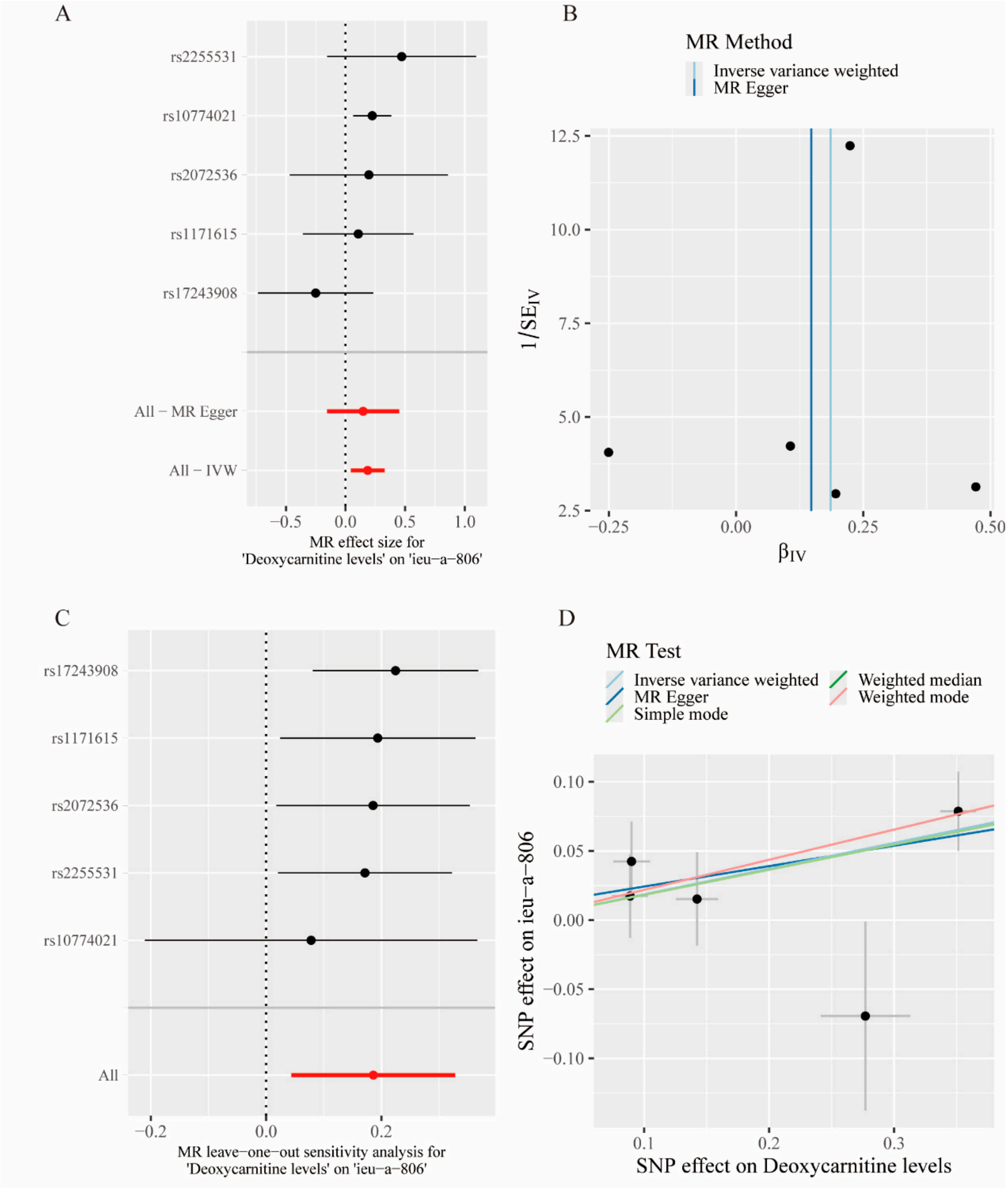

Figure S6. Various sensitivity analyses showed the robustness of the causal association between Deoxycarnitine and Autism Spectrum Disorder (ASD, GWAS ID: ieu-a-806).

(A) The forest plot showing no heterogeneity in causal effects amongst instruments. Each black point represents the effect size for ASD per standard deviation (SD) increase in the Deoxycarnitine produced using each of the SNPs as separate instruments, and red points showing the combined causal estimate using all SNPs together in a single instrument, using two different methods, including inverse variance weighted (IVW) and MR Egger. Horizontal lines denote 95% confidence intervals.

(B) Funnel plot showing the relationship between the causal effect of Deoxycarnitine on the ASD estimated using each individual SNP as a separate instrument against the inverse of the SE of the causal estimate. Vertical lines show the causal estimates using all SNPs combined into a single instrument for each of two different methods. Asymmetry in the funnel plot may

be indicative of violations of the instrumental variable (IV) through horizontal pleiotropy.
(C) Leave one out sensitivity analysis indicated that there are no instances where the exclusion of one particular SNP leads to dramatic changes in the overall result. Each black point represents the IVW MR method applied to estimate the causal effect of Deoxycarnitine on the ASD excluding that particular variant from the analysis. The red point depicts the IVW estimate using all SNPs.
(D) The scatter plot summarizing the MR estimates using 5 methods of statistics. The β-value with standard error (SE) is plotted to demonstrate effect estimate of each single nucleotide polymorphism (SNP) for causal association of Deoxycarnitine (x-axis) with the ASD (y-axis). The slope of each line represents the two-sample MR estimate (β-value) for the individual SNP. Error bar represents SE of effect size.
Metabolite is the exposure and ASD is the outcome.

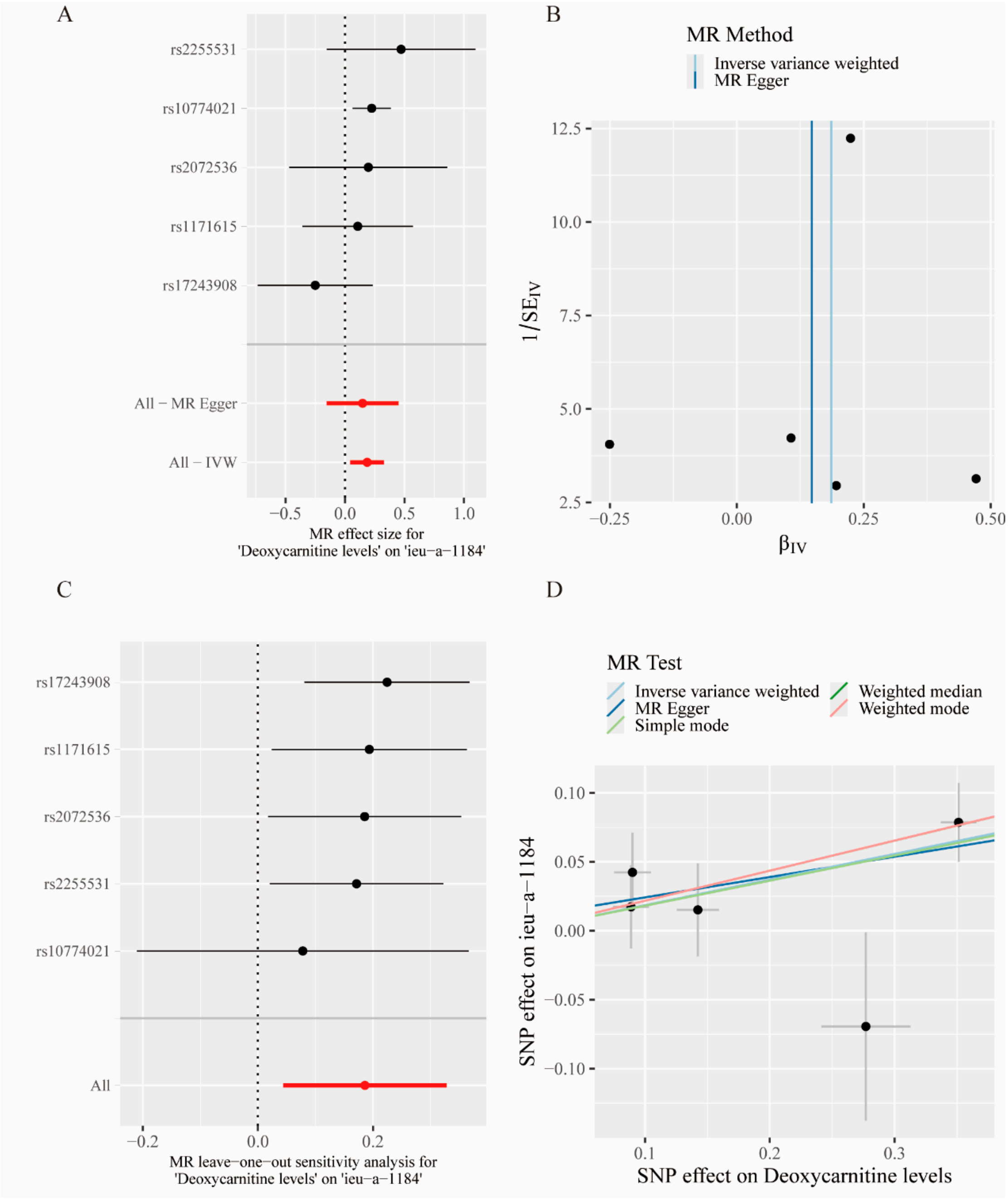

Figure S7. Various sensitivity analyses showed the robustness of the causal association between Deoxycarnitine and Autism Spectrum Disorder (ASD, GWAS ID: ieu-a-1184).

(A) The forest plot showing no heterogeneity in causal effects amongst instruments. Each black point represents the effect size for ASD per standard deviation (SD) increase in the Deoxycarnitine produced using each of the SNPs as separate instruments, and red points showing the combined causal estimate using all SNPs together in a single instrument, using two different methods, including inverse variance weighted (IVW) and MR Egger. Horizontal lines denote 95% confidence intervals.

(B) Funnel plot showing the relationship between the causal effect of Deoxycarnitine on the ASD estimated using each individual SNP as a separate instrument against the inverse of the SE of the causal estimate. Vertical lines show the causal estimates using all SNPs combined into a single instrument for each of two different methods. Asymmetry in the funnel plot may

be indicative of violations of the instrumental variable (IV) through horizontal pleiotropy.
(C) Leave one out sensitivity analysis indicated that there are no instances where the exclusion of one particular SNP leads to dramatic changes in the overall result. Each black point represents the IVW MR method applied to estimate the causal effect of Deoxycarnitine on the ASD excluding that particular variant from the analysis. The red point depicts the IVW estimate using all SNPs.
(D) The scatter plot summarizing the MR estimates using 5 methods of statistics. The β-value with standard error (SE) is plotted to demonstrate effect estimate of each single nucleotide polymorphism (SNP) for causal association of Deoxycarnitine (x-axis) with the ASD (y-axis). The slope of each line represents the two-sample MR estimate (β-value) for the individual SNP. Error bar represents SE of effect size.
Metabolite is the exposure and ASD is the outcome.

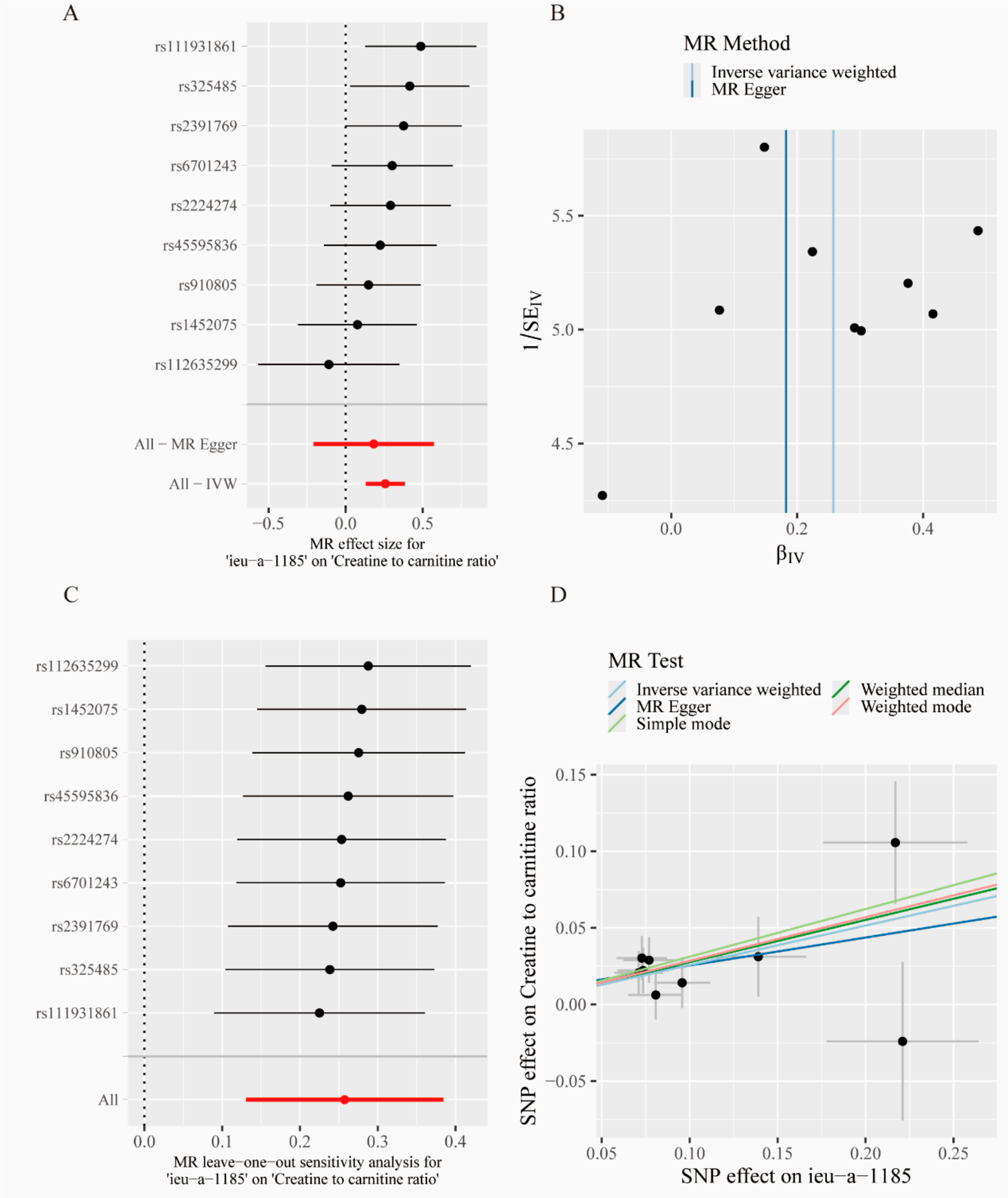

Figure S8. Various sensitivity analyses showed the robustness of the causal association between Autism Spectrum Disorder (ASD, GWAS ID: ieu-a-1185) and Creatine_to_carnitine.

(A) The forest plot showing no heterogeneity in causal effects amongst instruments. Each black point represents the effect size for ASD per standard deviation (SD) increase in the Creatine_to_carnitine produced using each of the SNPs as separate instruments, and red points showing the combined causal estimate using all SNPs together in a single instrument, using two different methods, including inverse variance weighted (IVW) and MR Egger. Horizontal lines denote 95% confidence intervals.

(B) Funnel plot showing the relationship between the causal effect of ASD on the Creatine_to_carnitine estimated using each individual SNP as a separate instrument against the inverse of the SE of the causal estimate. Vertical lines show the causal estimates using all SNPs

combined into a single instrument for each of two different methods. Asymmetry in the funnel plot may be indicative of violations of the instrumental variable (IV) through horizontal pleiotropy.

(C) Leave one out sensitivity analysis indicated that there are no instances where the exclusion of one particular SNP leads to dramatic changes in the overall result. Each black point represents the IVW MR method applied to estimate the causal effect of ASD on the Creatine_to_carnitine excluding that particular variant from the analysis. The red point depicts the IVW estimate using all SNPs.

(D) The scatter plot summarizing the MR estimates using 5 methods of statistics. The β-value with standard error (SE) is plotted to demonstrate effect estimate of each single nucleotide polymorphism (SNP) for causal association of ASD (x-axis) with the Creatine_to_carnitine (y-axis). The slope of each line represents the two-sample MR estimate (β-value) for the individual SNP. Error bar represents SE of effect size.

ASD is the exposure and metabolite is the outcome.

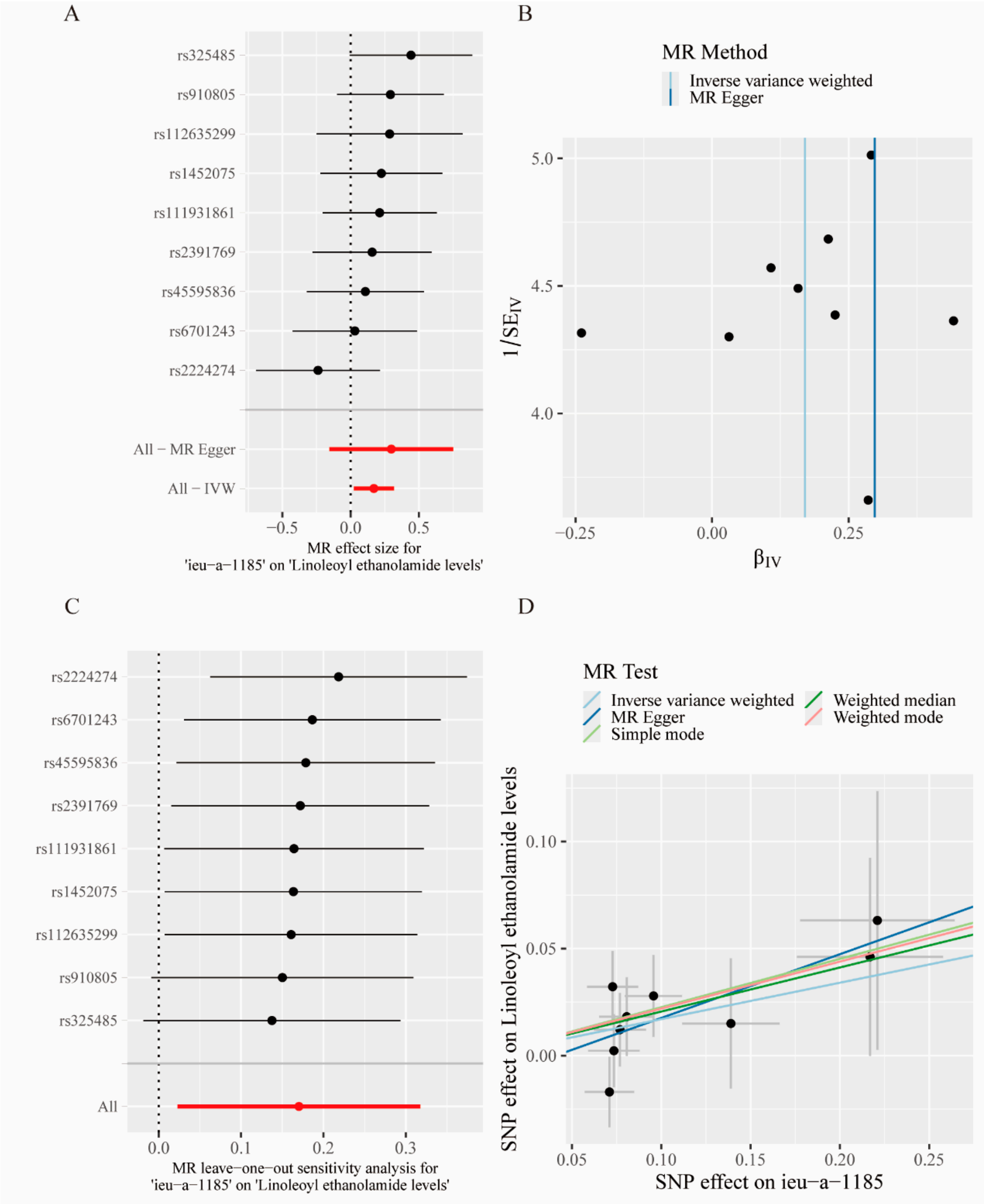

Figure S9. Various sensitivity analyses showed the robustness of the causal association between Autism Spectrum Disorder (ASD, GWAS ID: ieu-a-1185) and Linoleoyl_ethanolamide.

(A) The forest plot showing no heterogeneity in causal effects amongst instruments. Each black point represents the effect size for ASD per standard deviation (SD) increase in the Linoleoyl_ethanolamide produced using each of the SNPs as separate instruments, and red points showing the combined causal estimate using all SNPs together in a single instrument, using two different methods, including inverse variance weighted (IVW) and MR Egger. Horizontal lines denote 95% confidence intervals.

(B) Funnel plot showing the relationship between the causal effect of ASD on the Linoleoyl_ethanolamide estimated using each individual SNP as a separate instrument against

the inverse of the SE of the causal estimate. Vertical lines show the causal estimates using all SNPs combined into a single instrument for each of two different methods. Asymmetry in the funnel plot may be indicative of violations of the instrumental variable (IV) through horizontal pleiotropy.

(C) Leave one out sensitivity analysis indicated that there are no instances where the exclusion of one particular SNP leads to dramatic changes in the overall result. Each black point represents the IVW MR method applied to estimate the causal effect of ASD on the Linoleoyl_ethanolamide excluding that particular variant from the analysis. The red point depicts the IVW estimate using all SNPs.

(D) The scatter plot summarizing the MR estimates using 5 methods of statistics. The β-value with standard error (SE) is plotted to demonstrate effect estimate of each single nucleotide polymorphism (SNP) for causal association of ASD (x-axis) with the Linoleoyl_ethanolamide (y-axis). The slope of each line represents the two-sample MR estimate (β-value) for the individual SNP. Error bar represents SE of effect size.

ASD is the exposure and metabolite is the outcome.

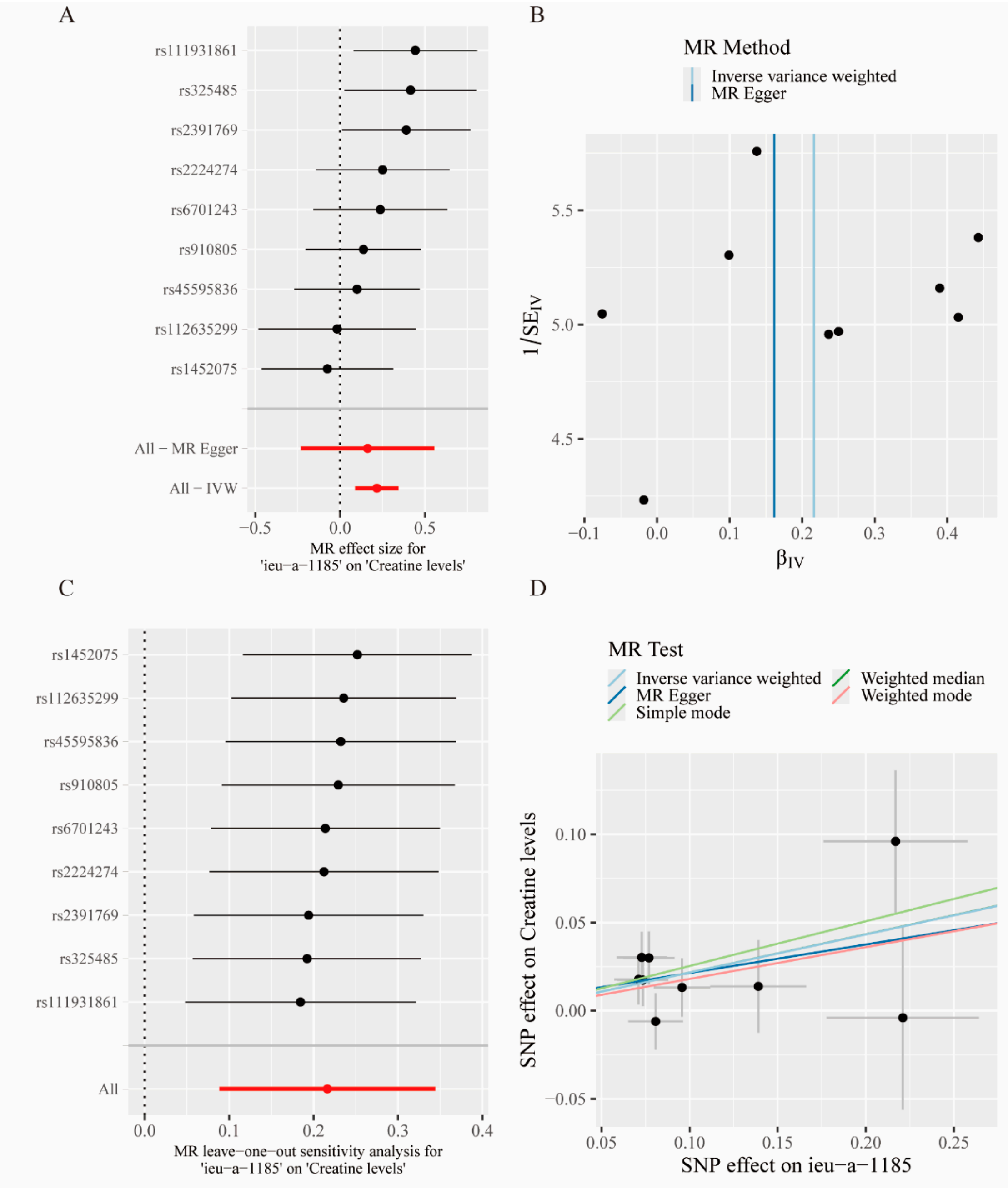

Figure S10. Various sensitivity analyses showed the robustness of the causal association between Autism Spectrum Disorder (ASD, GWAS ID: ieu-a-1185) and Creatine.

(A) The forest plot showing no heterogeneity in causal effects amongst instruments. Each black point represents the effect size for ASD per standard deviation (SD) increase in the Creatine produced using each of the SNPs as separate instruments, and red points showing the combined causal estimate using all SNPs together in a single instrument, using two different methods, including inverse variance weighted (IVW) and MR Egger. Horizontal lines denote 95% confidence intervals.

(B) Funnel plot showing the relationship between the causal effect of ASD on the Creatine estimated using each individual SNP as a separate instrument against the inverse of the SE of the causal estimate. Vertical lines show the causal estimates using all SNPs combined into a single instrument for each of two different methods. Asymmetry in the funnel plot may be

indicative of violations of the instrumental variable (IV) through horizontal pleiotropy.

(C) Leave one out sensitivity analysis indicated that there are no instances where the exclusion of one particular SNP leads to dramatic changes in the overall result. Each black point represents the IVW MR method applied to estimate the causal effect of ASD on the Creatine excluding that particular variant from the analysis. The red point depicts the IVW estimate using all SNPs.

(D) The scatter plot summarizing the MR estimates using 5 methods of statistics. The β-value with standard error (SE) is plotted to demonstrate effect estimate of each single nucleotide polymorphism (SNP) for causal association of ASD (x-axis) with the Creatine (y-axis). The slope of each line represents the two-sample MR estimate (β-value) for the individual SNP. Error bar represents SE of effect size.

ASD is the exposure and metabolite is the outcome.

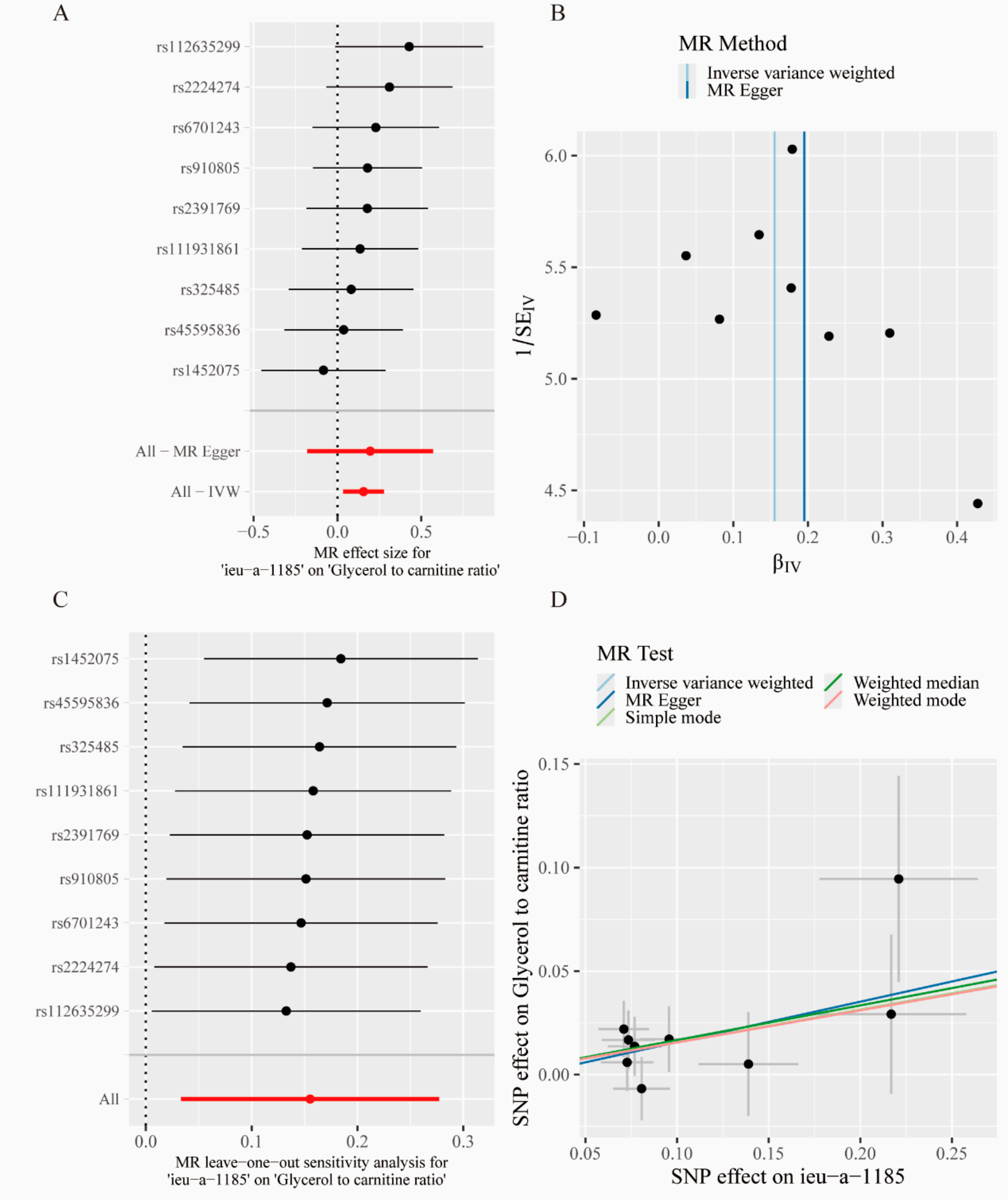

Figure S11. Various sensitivity analyses showed the robustness of the causal association between Autism Spectrum Disorder (ASD, GWAS ID: ieu-a-1185) and Glycerol to carnitine ratio.

(A) The forest plot showing no heterogeneity in causal effects amongst instruments. Each black point represents the effect size for ASD per standard deviation (SD) increase in the Glycerol to carnitine ratio. produced using each of the SNPs as separate instruments, and red points showing the combined causal estimate using all SNPs together in a single instrument, using two different methods, including inverse variance weighted (IVW) and MR Egger. Horizontal lines denote 95% confidence intervals.

(B) Funnel plot showing the relationship between the causal effect of ASD on the Glycerol to carnitine ratio. estimated using each individual SNP as a separate instrument against the inverse of the SE of the causal estimate. Vertical lines show the causal estimates using all SNPs combined into a single instrument for each of two different methods. Asymmetry in the funnel plot may be indicative of violations of the instrumental variable (IV) through horizontal pleiotropy.

(C) Leave one out sensitivity analysis indicated that there are no instances where the exclusion of one particular SNP leads to dramatic changes in the overall result. Each black point represents the IVW MR method applied to estimate the causal effect of ASD on the Glycerol to carnitine ratio. excluding that particular variant from the analysis. The red point depicts the IVW estimate using all SNPs.

(D) The scatter plot summarizing the MR estimates using 5 methods of statistics. The β-value with standard error (SE) is plotted to demonstrate effect estimate of each single nucleotide polymorphism (SNP) for causal association of ASD (x-axis) with the Glycerol to carnitine ratio. (y-axis). The slope of each line represents the two-sample MR estimate (β-value) for the individual SNP. Error bar represents SE of effect size.

ASD is the exposure and metabolite is the outcome.

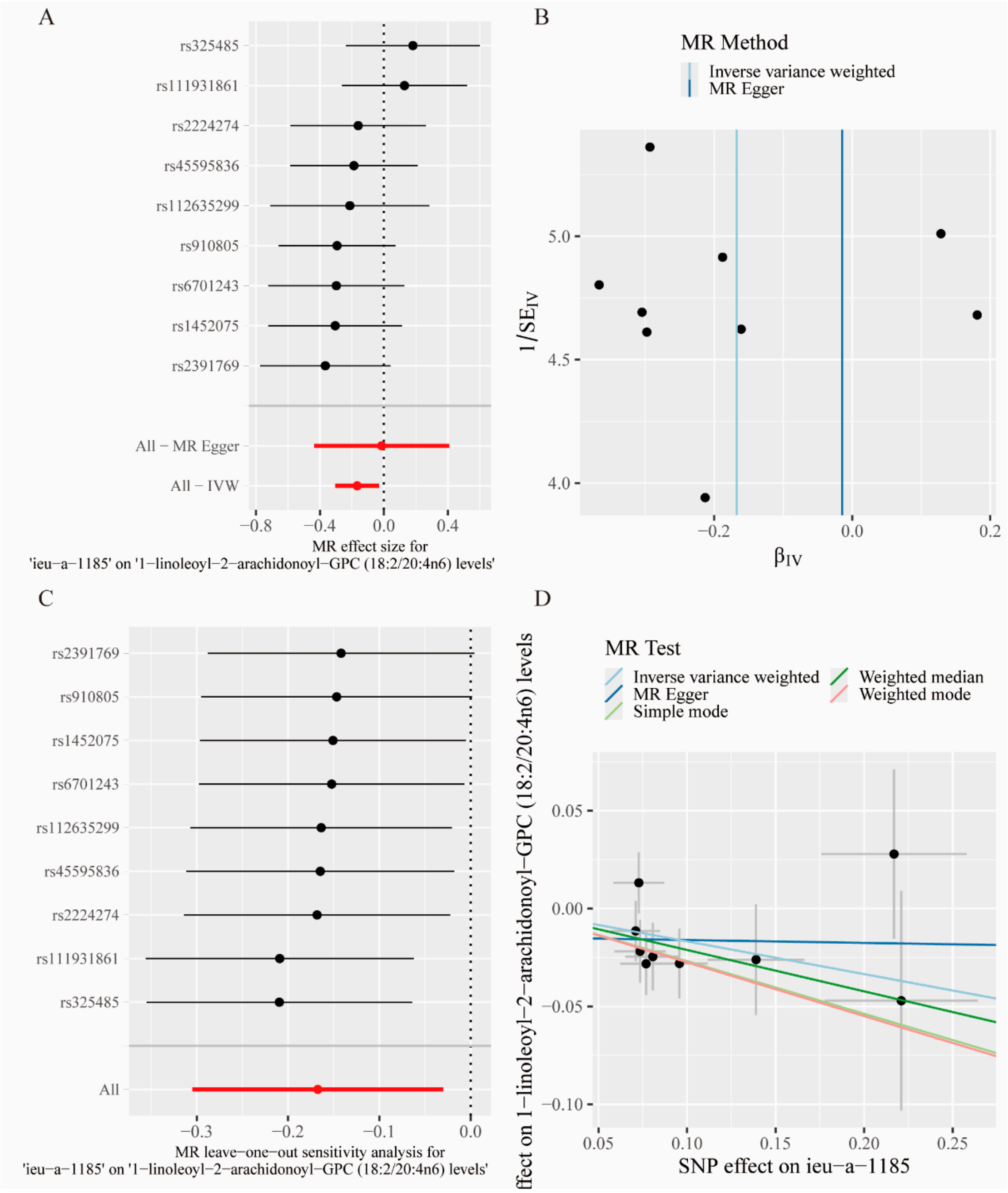

Figure S12. Various sensitivity analyses showed the robustness of the causal association between Autism Spectrum Disorder (ASD, GWAS ID: ieu-a-1185) and PC(18:2/20:4).

(A) The forest plot showing no heterogeneity in causal effects amongst instruments. Each black point represents the effect size for ASD per standard deviation (SD) increase in the PC(18:2/20:4) produced using each of the SNPs as separate instruments, and red points showing the combined causal estimate using all SNPs together in a single instrument, using two different methods, including inverse variance weighted (IVW) and MR Egger. Horizontal lines denote 95% confidence intervals.

(B) Funnel plot showing the relationship between the causal effect of ASD on the PC(18:2/20:4) estimated using each individual SNP as a separate instrument against the inverse of the SE of the causal estimate. Vertical lines show the causal estimates using all SNPs combined into a single instrument for each of two different methods. Asymmetry in the funnel plot may be

indicative of violations of the instrumental variable (IV) through horizontal pleiotropy.

(C) Leave one out sensitivity analysis indicated that there are no instances where the exclusion of one particular SNP leads to dramatic changes in the overall result. Each black point represents the IVW MR method applied to estimate the causal effect of ASD on the PC(18:2/20:4) excluding that particular variant from the analysis. The red point depicts the IVW estimate using all SNPs.

(D) The scatter plot summarizing the MR estimates using 5 methods of statistics. The β-value with standard error (SE) is plotted to demonstrate effect estimate of each single nucleotide polymorphism (SNP) for causal association of ASD (x-axis) with the PC(18:2/20:4) (y-axis). The slope of each line represents the two-sample MR estimate (β-value) for the individual SNP. Error bar represents SE of effect size.

ASD is the exposure and metabolite is the outcome.

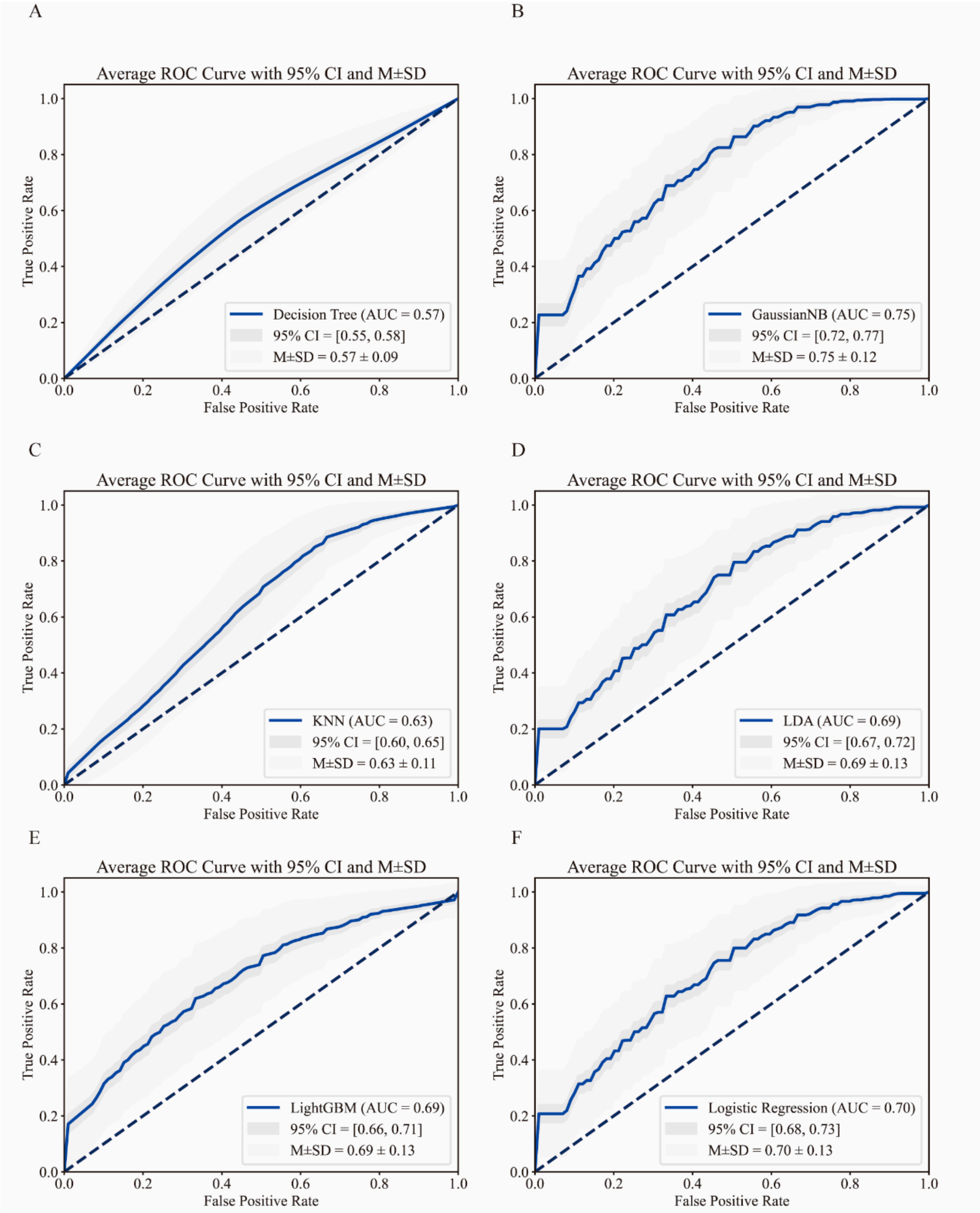

Figure S13. ROC curves and 95% CI and mean±SD for (A)Decision Tree, (B)GaussianNB, (C) KNN, (D)LDA, (E)LightGBM, (F)Logistic Regression.

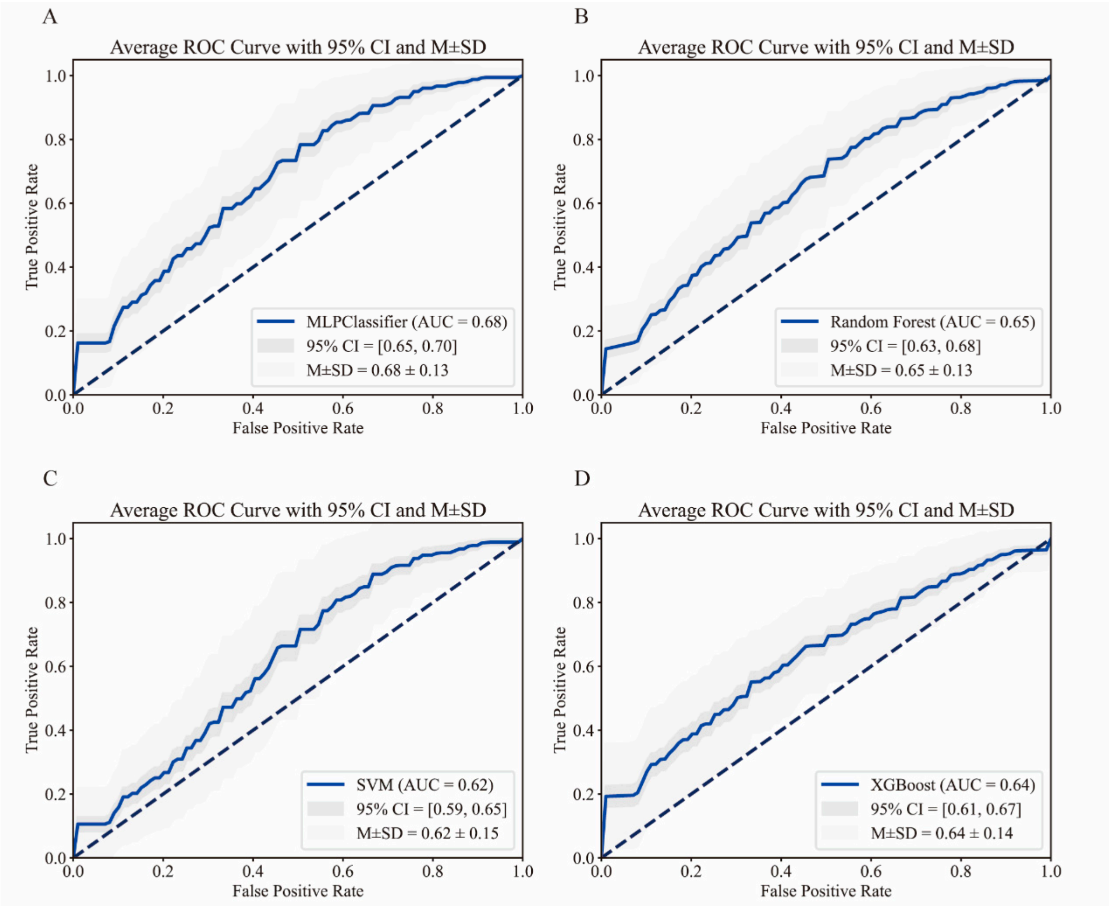

Figure S14. ROC curves and 95% CI and mean±SD for (A)MLP, (B)Random Forest, (C)SVM, (D)XGBoost.
